# DEPDC1 as a metabolic target regulates glycolysis in renal cell carcinoma through AKT/mTOR/HIF1α pathway

**DOI:** 10.1038/s41419-024-06913-1

**Published:** 2024-07-27

**Authors:** Si-chen Di, Wen-jin Chen, Wei Yang, Xiang-min Zhang, Ke-qin Dong, Yi-jun Tian, Ye Sun, Cheng Qian, Jia-xin Chen, Zi-chang Liu, Zi-xuan Gong, Jian Chu, Wang Zhou, Xiu-wu Pan, Xin-gang Cui

**Affiliations:** 1 Department of Urology, Xinhua Hospital, School of Medicine, Shanghai Jiaotong University, Shanghai, China; 2 Department of Urology, Third Affiliated Hospital of the Second Military Medical University, Shanghai, China; 3 Department of Urology, Shanghai Baoshan Luodian Hospital, Shanghai, China; 4 Department of Urology, Chinese PLA General Hospital of Central Theater Command, Wuhan, China; 5 Department of Urology, Tongji Hospital, School of Medicine, Tongji University, Shanghai, China; 6 Department of Urology, Taian 88 Hospital, Taian, Shandong China; 7 Department of Urology, Shanghai Pudong New Area Gongli Hospital, Shanghai, China

**Keywords:** Cancer metabolism, Renal cell carcinoma

## Abstract

Renal cell carcinoma (RCC) is considered a “metabolic disease” characterized by elevated glycolysis in patients with advanced RCC. Tyrosine kinase inhibitor (TKI) therapy is currently an important treatment option for advanced RCC, but drug resistance may develop in some patients. Combining TKI with targeted metabolic therapy may provide a more effective approach for patients with advanced RCC. An analysis of 14 RCC patients (including three needle biopsy samples with TKI resistance) revealed by sing-cell RNA sequencing (scRNA-seq) that glycolysis played a crucial role in poor prognosis and drug resistance in RCC. TCGA-KIRC and glycolysis gene set analysis identified DEPDC1 as a target associated with malignant progression and drug resistance in KIRC. Subsequent experiments demonstrated that DEPDC1 promoted malignant progression and glycolysis of RCC, and knockdown DEPDC1 could reverse TKI resistance in RCC cell lines. Bulk RNA sequencing (RNA-seq) and non-targeted metabolomics sequencing suggested that DEPDC1 may regulate RCC glycolysis via AKT/mTOR/HIF1α pathway, a finding supported by protein-level analysis. Clinical tissue samples from 98 RCC patients demonstrated that DEPDC1 was associated with poor prognosis and predicted RCC metastasis. In conclusion, this multi-omics analysis suggests that DEPDC1 could serve as a novel target for TKI combined with targeted metabolic therapy in advanced RCC patients with TKI resistance.

## Introduction

By 2021, while overall cancer death rates are declining, progress in cancer prevention is lagging, with rates continuing to increase for six of the top 10 cancers, including renal cell carcinoma, which continues to increase at a rate of 1.5% per year [1]. Renal cell carcinoma (RCC) constitutes around 90% of all renal carcinoma cases [2]. Approximately one-third of renal cell carcinoma patients were already diagnosed with advanced metastatic renal cell carcinoma at the initial diagnosis [3]. Even though some early-stage patients underwent surgical treatment, 30–40% of patients still developed metastasis or postoperative recurrences [4]. Currently, tyrosine kinase inhibitor (TKI) has served as the first-line therapy for advanced RCC, demonstrating substantial enhancements in overall survival (OS) and progression-free survival (PFS) in patients with advanced RCC [5]. Nonetheless, patients undergoing TKI therapy are susceptible to developing TKI resistance, thus diminishing the therapeutic efficacy [5]. Hence, there is an urgent need to investigate novel treatment modalities to counter TKI resistance and enhance the therapeutic effectiveness and improve the prognosis of patients with advanced RCC.

Given the presence of unique von Hippel-Lindau (VHL) gene mutation in kidney tumors causing various metabolic anomalies, such as glucose metabolism, lipid metabolism, and amino acid metabolism, RCC is commonly referred to as a “metabolic disease” [6]. Current research demonstrates that “metabolism” is a key characteristic of cancer, and all histologies of RCC display increases in metabolic activity with disease progression. In particular, one common feature is the invocation of aerobic glycolysis, known as the “Warburg effect” [7, 8]. Mammalian target of rapamycin (mTOR) stands as the primary metabolic target for treating RCC, knowing that multiple studies have illustrated its role in regulating HIF1α translation and glycolytic activity [6]. Nonetheless, given the propensity of advanced RCC patients to develop resistance to mTOR inhibitors and TKIs, exploration of new targets in glucose metabolism within RCC to counter drug resistance in this patient population represents a promising avenue for future research.

Recently, a ccRCC proteomic study reported that the PI3K/AKT/mTOR pathway was the resistance mechanism of sunitinib treatment in ccRCC patients, and the Warburg effect derived from VHL mutations can further enhance TKI resistance in ccRCC [9]. In the present study, we identified glycolysis as a key factor contributing to poor prognosis and drug resistance in RCC by using scRNA-seq analysis, and found that the glycolysis-related gene DEP domain containing 1 (DEPDC1) played a role in malignant progression and drug resistance of RCC, and may regulate RCC glycolysis through AKT/mTOR/HIF1α pathway. However, in previous studies, DEPDC1 has been little studied in tumor glycolysis. As only a glycolytic-related gene, DEPDC1 can regulate the malignant progression of oral squamous cell carcinoma through the WNT/β-Catenin pathway, and as one of the prognostic risk predictors of hepatocellular carcinoma [10, 11]. In our study, DEPDC1 was identified as a promising new therapeutic target for patients with advanced RCC, especially when it was combined with TKIs and targeted metabolism therapies.

## Materials and methods

### Patients and sample collection

All experimental procedures were approved by the ethical review board of Xinhua Hospital (Shanghai, China), and written informed consent was obtained from all included patients. Fourteen scRNA-seq samples were obtained from surgical resection tissues or needle biopsy tissues of patients with pathologically confirmed RCC. Data details are shown in Table 1. The two tissue microarrays (TMA30, *n* = 28 and TMA2021, *n* = 70) were obtained from 98 patients from Xinhua Hospital. The clinical information is detailed in Table 2, S1 and S2. TCGA sample data came from UCSC Xena (https://xena.ucsc.edu/), including RNA-seq, somatic mutation and clinical data. The clinical information is detailed in Table S3.

**Table 1:** Clinical information of RCC patients in scRNA-seq analysis.

Patient ID	Gender	Age	Grade	Stage	Treatment
C1	Female	62	1	III	Naive
C2	Male	46	2	I	Naive
C3	Female	58	2	I	Naive
C4	Male	65	3	I	Naive
C5	Male	59	3	IV	Naive
C6	Male	70	2	II	Naive
C7	Female	71	2	II	Naive
C8	Female	49	3	III	Naive
C9	Male	65	3	IV	Naive
C10	Female	71	2	IV	Naive
C11	Female	49	3	III	Naive
C12	Male	63	3	IV	Sunitinib, non-response
C13	Male	57	4	IV	Pazopanib, non-response
C14	Male	39	4	IV	Pazopanib, non-response

**Table 2:** Clinical characteristics of patients according to DEPDC1 expression in TMA (*n* = 98).

Characteristics	DEPDC1 in TMA		Sum (*n* = 98)	*P* value
	High expression (*n* = 36)	Low expression (*n* = 62)		
Diagnosis age				0.0088
<60	14	41	55	
≥60	22	21	43	
Gender				0.2813
Male	23	46	69	
Female	13	16	29	
Fuhrman grade				0.0037
G1-2	25	57	82	
G3-4	11	5	16	
TNM stage				0.0019
I–II	29	61	90	
III–IV	7	1	8	
Any metastases or recurrence				<0.0001
Yes	21	6	27	
No	15	56	71	
Overall survival				<0.0001
Dead	22	9	31	
Alive	14	53	67	

### scRNA-seq and data processing

Single-cell suspension and droplet sequencing was performed according to the manufacturer’s protocol and our previous work [12]. Data quality control was performed using Seurat (version 3.0.1) [13]. Markers for each major cell cluster were identified by the “FindAllMarker” function. Cell type markers were obtained from CellMarker website [14] and previous studies [15, 16].

### InferCNV and cell malignancy evaluation

Copy number variations (CNVs) were investigated by scRNA-seq analysis using pipeline parameters of the InferCNV package. Each cell was scored based on the extent of the CNV signal, which was defined according to the previous work [12, 17]. Finally, cells with CNV signal greater than 0.05 and CNV correlation coefficient greater than 0.5 were defined as malignant cells, and cells lower than these two thresholds were defined as non-malignant cells.

### High-dimensional weighted correlation network analysis (hdWGCNA)

The scRNA-seq data obtained were analyzed by hdWGCNA. Genes that were expressed in at least 5% cells were selected to construct the hdWGCNA object, which was then transformed into a Metacells object. A co-expression network was constructed for subsequent analysis (soft threshold = 4, max Block Size = 30, min Module Size = 30). All standard downstream analyses were conducted according to the official pipeline, which can be found at https://smorabit.github.io/hdWGCNA/articles/basic_tutorial.html (accessed on 1 January 2023) [18]. First, soft threshold calculation was performed to obtain the optimal soft threshold, and then the similarity matrix was obtained by calculating the Pearson correlation coefficient between genes. Then, the correlation between different modules and different clinical stages was calculated by the harmony-corrected module signature genes (hME), and the correlation between genes within the module and the module itself was calculated by the membership module (kME).

### Gene set enrichment analysis (GSEA)

Malignant cells in scRNA-seq, TCGA samples, and RNA-seq samples were divided into DEPDC1^high^ group and DEPDC1^low^ group. GSEA was used to determine which gene sets were enriched in subgroup comparisons. Only a *p* value of a gene less 0.05 was considered as the target of interest. The annotation gene sets included GO_BP, GO_MF, GO_CC, KEGG, HALLMARK, REACTOME and WikiPathways from Molecular Signatures Database (MSigDB) [19].

### Public database analysis

Two hundred glycolysis genes were obtained from MSigDB in Table S4 [19]. Using bioinformatics, a Venn diagram was created [20]. The mRNA expression of DEPDC1 in pan-cancer was obtained from Timer 2.0 [21]. OS of survival curves for DEPDC1 was obtained by Kaplan-Meier Plotter [22]. OS and Disease-free survival (DFS) of survival curve for DEPDC1, differential expression of key glycolysis enzymes in KIRC, and the correlation between key glycolysis enzymes and DEPDC1 were obtained through GEPIA2 [23]. The half inhibitory concentration (IC_50_) of DEPDC1 against sunitinib and pazopanib was obtained from GDSC database (https://www.cancerrxgene.org/). Expression levels of DEPDC1 between tumors and normal tissues, as well as different nodal metastasis statuses, tumor grades, and cancer stages in KIRC, were retrieved using UALCAN [24].

### Cell culture

Human renal tubular epithelial cells (HK-2), 786-O, 769-P, OS-RC-2, A498 and ACHN were obtained from the Cell Bank of the Chinese Academy of Sciences (Shanghai, China). Cells were cultured with 1640 or DMEM + 10% fetal bovine serum (FBS) + 1% penicillin at 37°C and 5% CO_2_.

Sunitinib-resistant 786-O (786-O-R) cell lines were continuously exposed to increasing doses of sunitinib (MCE, HY-10255A) for about 12 weeks. The starting dose was 5 µM for 4 weeks, which was increased to 10 µM for additional 4 weeks, to 15 µM for last 4 weeks [25]. 786-O-R cell lines were finally cultured in RPMI-1640 supplemented with 10% (v/v) FBS and 10 µM Sunitinib [26].

### Lentiviral gene tool establishment and gene knockdown

The overexpression or shRNA lentivirus DEPDC1 (OE-DEPDC1 and sh-DEPDC1) was synthesized by OBiO technology (Shanghai, China) and the siRNA of DEPDC1 or negative control siRNA was synthesized by IBSBIO (Shanghai, China) using the sequences (Table S5). The OS-RC-2, 786-O and 786-O-R cell lines were constructed using lentivirus to stably knock down DEPDC1. The A498 and ACHN cell lines were constructed using lentivirus to stably overexpress DEPDC1. Lipofectamine 3000 reagent (L3000015, Invitrogen) was used for siRNA transfection according to the manufacturer’s protocol.

### Quantitative real-time PCR (qRT-PCR)

Total RNA was extracted using TRIZOL (Invitrogen, USA) and reversed transcribed into cDNA. Gene transcripts were quantitated by SYBR Green Real-Time PCR Master Mix (QPK201, Japan), and then normalized to the β-actin expression. The sequences of primers are shown in Table S6.

### Western blot

Cell samples were lysed in RIPA buffer (Beyotime, China) mixed with the loading buffer. The obtained proteins were separated on SDS/PAGE gel and then transferred into PVDF membranes. Proteins were incubated with specific primary antibodies and proper secondary antibodies. The blots were detected by ECL system. The following antibodies were used: anti-DEPDC1 (Abcepta, AP5428a), anti-β-actin (ABclonal, AC026), anti-Phospho-AKT(Ser473) (Cell Signaling Technology, 4060), anti-AKT(pan) (C67E7) (Cell Signaling Technology, 4691), anti-Phospho-mTOR (Ser2448) (Cell Signaling Technology, 5536), anti-mTOR (7C10) (Cell Signaling Technology, 2983), anti-HIF1α (proteintech, 20960), anti-HK-2 (proteintech, 22029), anti-PKM2 (proteintech, 15822), and anti-LDHA (proteintech, 19987).

### Cell function assays

For cell proliferation assessment, the proliferation of RCC cells under specified conditions was detected with CCK8 kit (Dojindo Kumamoto, Japan) according to the manufacturer’s instructions. The media were replaced with fresh ones before testing. 10 μl CCK-8 was added to each well, and the samples were cultured at 37 °C for 2 h. OD values were measured at 450 nm absorbance using a microplate reader. For migration and invasion experiments, transwell chambers (Corning, USA) were used without or with Matrigel (BD Biosciences, USA). Cells were seeded in the upper chamber with medium lacking FBS, while the lower chamber was plated with medium containing FBS. After 24-h seeding, cells in the lower chamber were fixed using 4% paraformaldehyde Fix Solution (Sangon Biotech, Shanghai), stained with crystal violet (Sangon Biotech, Shanghai), and then scanned at ×200 magnification.

### Glycolytic metabolite assays

Cells were seeded in a 6-well plate and cultured for 24 h. Then the medium was collected and tested for glucose, lactate, and pyruvate using the glucose assay kit, lactate assay kit, and pyruvate assay kit (Nanjing Jian Cheng Bioengineering Institute, China) following the manufacturer’s protocols.

### Animal experiments

All animal experimental procedures were approved by Xinhua hospital Animal Care and Use Committee. Approximately 5 × 10^6^ OE-DEPDC1 A498 cells or control A498 cells were injected subcutaneously into the upper right hind leg of the mice. Body weight and tumor size were measured weekly, and at the 7th week, tumors were removed, weighed and measured for volume. The tissue was then fixed in a 10% buffered formalin solution for subsequent immunohistochemical (IHC) analysis.

### Bulk RNA sequencing (RNA-seq)

One milliliter TRIzol was separately added to 5 × 10^6^ 786-O cells (sh-NC or sh-DEPDC1) to isolate total RNA. Samples were then immediately stored on dry ice. Subsequently, the samples were subjected to RNA quality control testing and sequenced using the Illumina NovaSeq 6000 sequencer from Cosmos Wisdom (Hangzhou, China). Data obtained from sequencing were analyzed using R software.

### Non-targeted metabolomics sequencing

1 × 10^7^ 786-O (sh-NC and sh-DEPDC1) and 1 × 10^7^ 786-O-R (sh-NC and sh-DEPDC1) cells were prepared, washed twice with PBS buffer, transferred to a 1.5 ml EP tube, soaked in liquid nitrogen for ≥ 5 min to quench, and stored at −80 °C for later use. Then sequencing was performed according to the method of Cosmos Wisdom (Hangzhou, China), and the sequencing data were analyzed using R software.

### IHC and H-score

IHC was conducted following the previous protocol [12], using rabbit anti-DEPDC1 (Abcepta, AP5428a) for staining. The IHC results were evaluated using H-score. The scoring system categorized results as negative, low positive, positive, and high positive. The H-score = 0 * the percentage of negative cell nucleus + 1 * the percentage of low-positive cell nucleus + 2 * the percentage of positive cell nucleus + 3 * the percentage of high-positive cell nucleus, which ranges from 0 to 300 [12].

### Statistical analysis

Statistical differences between numerical data (mean ± SD) were calculated by Student’s *t* test (two-tailed). Categorical variables were analyzed using the chi-square test. Univariate Cox regression analysis was performed using SPSS 22.0 (IBM corporation). Survival curves were drawn using GraphPad Prism 9.5 (GraphPad Software, Inc.), and receiver operating characteristic (ROC) analysis was performed to obtain the critical value of H-score and AUC. Nomogram analysis was conducted by “foreign” (version 0.8–78) and “rms” (version 6.0.1) packages for establishing the risk prediction model. All experiments were performed independently at least three times.

## Results

### scRNA-seq identifies glycolysis as a key module leading to poor prognosis and drug resistance in RCC

**Fig. 1:**
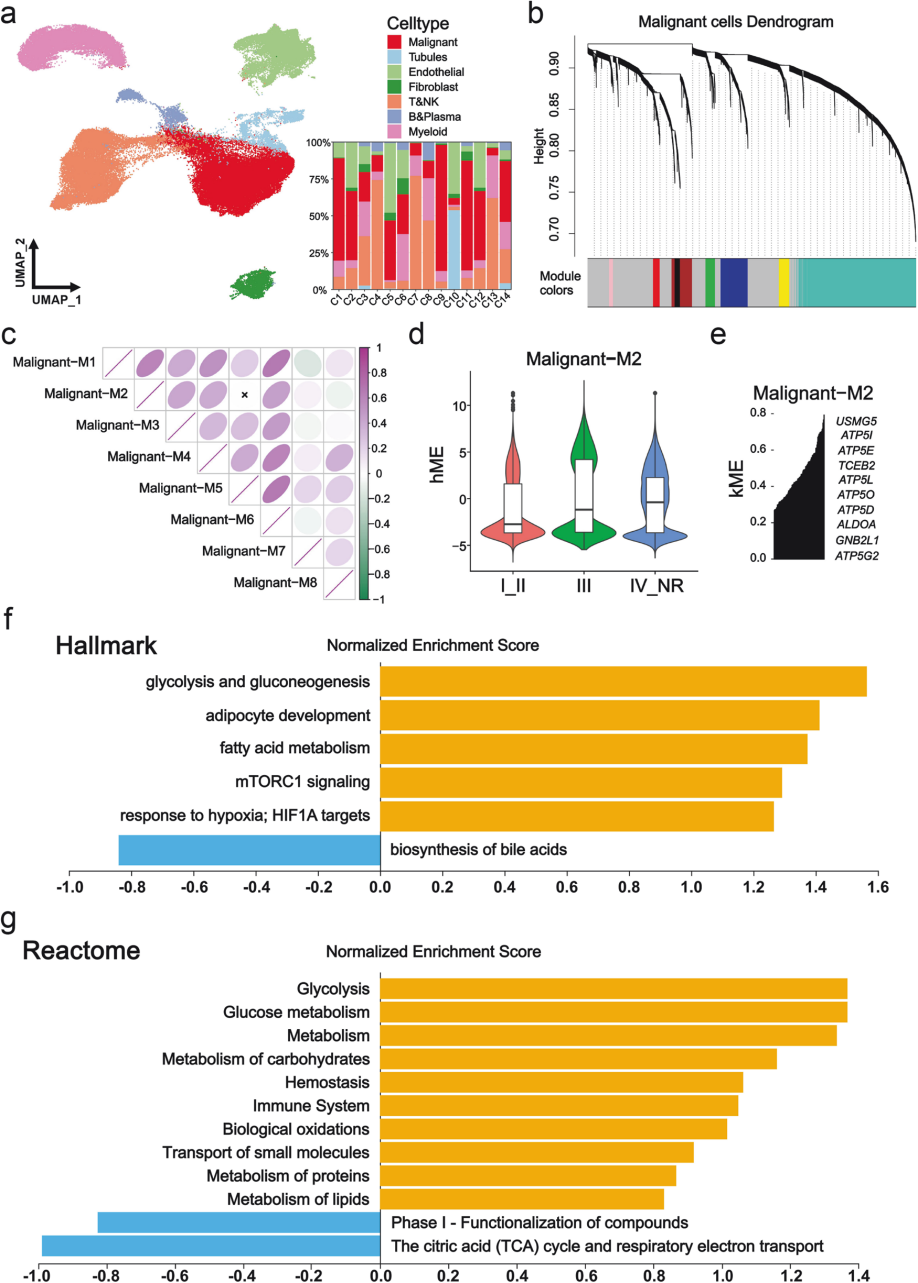
scRNA-seq discovered a key module for poor prognosis and drug resistance in RCC. **a** UMAP plot showing all cells and composition proportions of 14 patients, including malignant, tubules, endothelial, fibroblasts, T&NK, B&Plasma, and Myelold cells. **b** Dendrogram visualizing the hierarchical structure of the co-expression network, where each module color represents a distinct module. **c** Inter-module correlation analysis, showing the strength of correlation between each module and all other modules. **d** hME showing the module characteristic values of gene modules in different clinical stages. **e** kME showing the correlation between core genes and gene modules. **f** Hallmark enrichment analysis showing a positive correlation of M2 module genes with glycolysis, mTORC1 signaling and other related pathways. **g** Reactome enrichment analysis showing a positive correlation of M2 module genes with glycolysis, glucose metabolism and other related pathways, and a negative correlation with TCA cycle. *p < 0.05, **p < 0.01, ***p < 0.001.

To investigate the relationship between clinical malignancy and glycolysis in RCC, scRNA-seq analysis was conducted on primary tumors from 14 RCC patients, including 3 needle biopsy samples with TKI resistance (Table 1). Quality control measures involved analyzing 87,770 cells from the 14 patients, which were clustered into 7 major cell types, involving 33,557 malignant cells, renal tubular cells, endothelial cells, fibroblasts, T cells and NK cells, B cells and plasma cells, and myeloid cells (Table S7). The cell composition ratio for each patient is presented in Fig. 1a. Using the Test Soft Powers function in the hdWGCNA package, different soft thresholds were tested to find a suitable value that gives the constructed co-expression network a scale-free network structure. Ultimately, 4 was selected as the optimal soft threshold, making the topology of the network most consistent with the actual biological relationships (Fig. S1a). Subsequently, we explored the relationships between gene modules in the co-expression network by measuring gene expression similarity, calculating the topological overlap matrix, and performing hierarchical clustering analysis. The hierarchical structure of the co-expression network was visualized using a dendrogram (Fig. 1b). Furthermore, correlation analysis demonstrated the strength of correlations between each module and all other modules (Fig. 1c). The module characteristic values at different clinical stages were determined via the harmony-corrected module signature genes (hME), revealing an increase only in the hME value of the M2 module with clinical stage (Figs. 1d, S1b and Table S8). Next, we obtained genes with representative expression patterns in each module (module eigengenes) and calculated the correlation of each gene with the module eigenvector (module connectivity, kME). We visualized the relationships between genes within modules using a connectivity plot (Figs. 1e, S1c and Table S9). Further analysis involved GSEA functional enrichment of all genes within the M2 module, indicating a positive correlation with glycolysis, mTORC1 signaling, HIF1α targeting, and other pathways, while showing a negative correlation with the citric acid (TCA) cycle and respiratory electron transport (Fig. 1f, g). These findings suggest that glycolysis played a critical role in driving poor prognosis and drug resistance in RCC.

### Glycolysis-related gene DEPDC1 is associated with malignant progression and drug resistance in KIRC

**Fig. 2:**
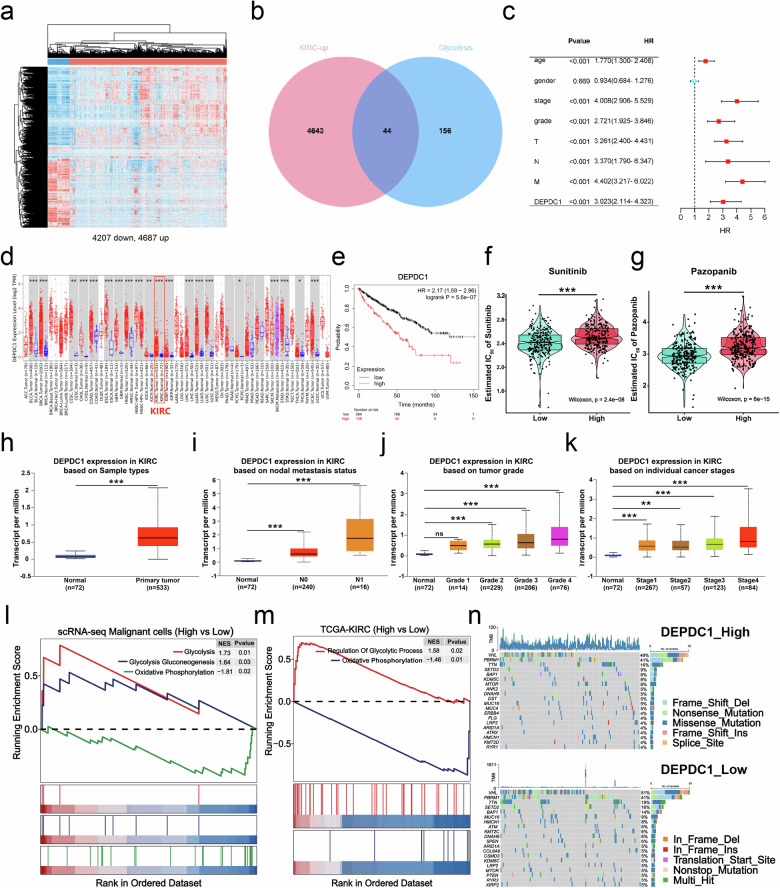
Glycolysis-related gene DEPDC1 is associated with malignant progression and drug resistance in KIRC. **a** Heat map demonstrating the results of the differential expression analyses using the TCGA-KIRC dataset, including 4687 upregulated DEGs and 4207 down-regulated DEGs. **b** Venn diagram illustrating the intersection of 4687 upregulated DEGs and 200 GRGs. **c** Univariate Cox regression analysis of DEPDC1 and clinical information. **d** Timer 2.0 database demonstrating the expression level of DEPDC1 in pan-cancer. **e** Analysis of the K–M survival curve of DEPDC1 high and low groups in TCGA-KIRC (*n* = 530). **f**, **g** The GDSC database provides IC_50_ predictions for sunitinib and pazopanib in Endo_p high and low risk score groups. **h–k** UALCAN database offering insights into the expression levels of DEPDC1 in tumors and normal tissues, different nodal metastasis statuses, different tumor grades, and different cancer stages. **l**, **m** The GSEA analysis of high DEPDC1 expression vs low DEPDC1 expression in malignant cells by scRNA-seq and TCGA-KIRC. **n** The oncoprint of conventional marker genes of RCC with alterations in DEPDC1 high and low groups, where tumor mutation burden is represented for individual samples as a bar chart above the oncoprint. *p < 0.05, **p < 0.01, ***p < 0.001. DEPDC1 DEP domain-containing protein 1, KIRC Kidney clear cell carcinoma, TCGA The Cancer Genome Atlas.

Knowing that glycolysis played a crucial role in the poor prognosis and drug resistance in RCC, we conducted further investigations into the connection between RCC and glycolysis. Through a differential expression analysis of TCGA-KIRC, we identified 4687 upregulated differentially expressed genes (DEGs) and 4207 down-regulated DEGs (Fig. 2a). Subsequently, we focused on identifying glycolysis-related biomarkers that drive RCC progression by intersecting the 4687 upregulated DEGs with the glycolysis gene set. As a result, 44 glycolysis-related genes (GRGs) were identified (Fig. 2b and Table S4). The univariate Cox regression analysis based on the clinical information highlighted the gene DEPDC1 as having the highest risk ratio (HR = 3.023, *p* < 0.001) (Fig. 2c). The comprehensive results of univariate Cox regression analysis are shown in Fig. S2a. Timer 2.0 database analysis revealed that DEPDC1 was expressed at higher levels in tumor tissues compared to normal tissues, particularly in KIRC (Fig. 2d). Furthermore, high DEPDC1 expression was linked to poor prognosis in KIRC patients (Fig. 2e), a finding corroborated by the GEPIA2 database (Fig. S2b, c). Exploring the relationship between DEPDC1 expression and TKIs for first-line treatment of advanced RCC using the Genomics of GDSC database demonstrated that the sensitivity to sunitinib and pazopanib was enhanced in the low DEPDC1 expression group, as evidenced by lower IC_50_ of response compared to the high-expression group (Fig. 2f, g). Additionally, the UALCAN database revealed that high DEPDC1 expression correlated with nodal metastasis, high tumor grade, and advanced cancer stage in KIRC patients (Fig. 2h–k). To investigate the link between the glycolysis-related gene DEPDC1 and glycolysis in RCC, we categorized all malignant cells from Fig. 1a and patients from TCGA-KIRC into high and low groups based on DEPDC1 expression levels. GSEA revealed a positive correlation between high DEPDC1 expression and glycolysis, as well as a negative correlation with oxidative phosphorylation (Fig. 2l, m). Finally, we assessed somatic alterations in TCGA patients based on the mean expression of DEPDC1 (high group, *n* = 160; low group, *n* = 160). Genetic changes of previous studies on RCC are shown in Fig. 2n. The BAP1, MUC16 and MTOR were presented differences in DEPDC1 high and low groups (Fig. 2n). These results suggest that DEPDC1, a glycolysis-related gene could serve as a prognostic marker for poor prognosis and aggressive disease progression in KIRC patients, potentially influencing drug resistance and enhancing glycolysis in RCC.

### DEPDC1 enhances the proliferation, migration, invasion, and subcutaneous tumor formation ability of RCC cells

**Fig. 3:**
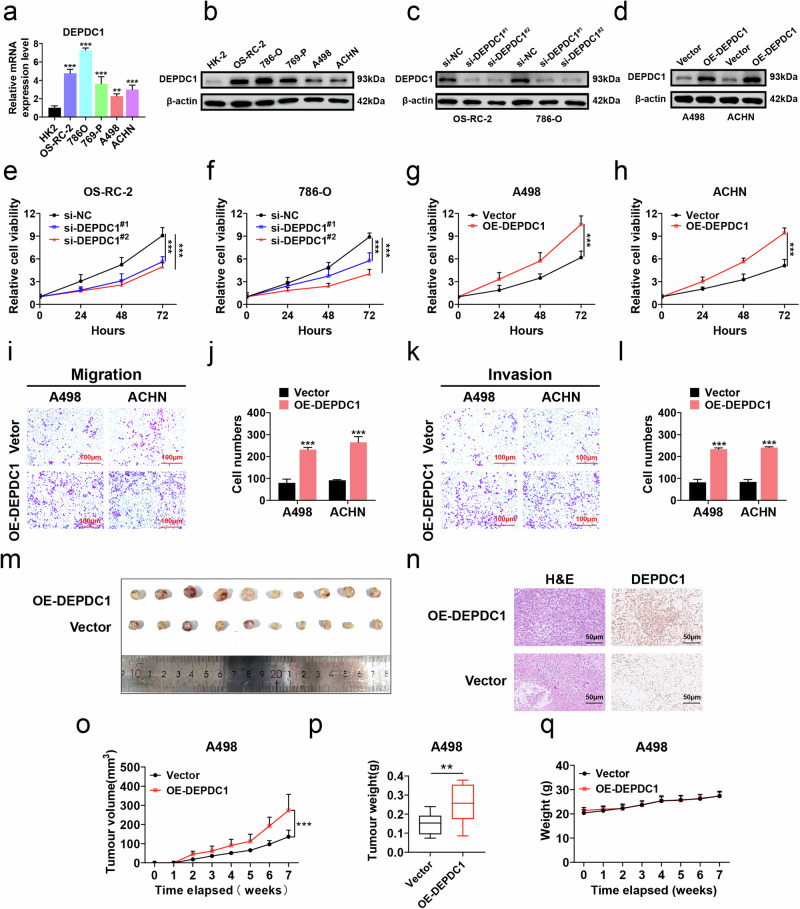
DEPDC1 promotes malignant progression of RCC. **a**, **b** qRT-PCR and western blot revealing differential expression of DEPDC1 at the mRNA and protein levels in various RCC cell lines (OS-RC-2, 786-O, 769-P, A498, ACHN) compared to HK-2 control group. **c** Western blot showing the knockdown efficiency of DEPDC1 in OS-RC-2 and 786-O cells. **d** Western blot showing the overexpression efficiency of DEPDC1 in A498 cells and ACHN cells. **e**, **f** Cell proliferation assay indicating the increased growth rate and proliferative activity of OS-RC-2 and 786-O cells in DEPDC1 knockdown group (si-DEPDC1^#1^ and si-DEPDC1^#2^) compared with si-NC control group. **g**, **h** Cell proliferation assay showing changes in proliferation activity of A498 cells and ACHN cells in DEPDC1 overexpression group (OE-DEPDC1) compared with the Vector control group. **i**–**l** Transwell assay showing changes in the number of migration and invasion cells in A498 and ACHN cells in OE-DEPDC1 group compared with Vector control group, scale bar = 100 μm. **m** Representative images of xenografts collected from mice receiving Vector or OE-DEPDC1 A498 cells (*n* = 10). **n** IHC staining of DEPDC1 in xenograft tumor cell nuclei in Vector and OE-DEPDC1 groups. **o**–**q** Tumor growth curve, weight comparison, and body weight changes of nude mice were collected from mice receiving Vector or OE-DEPDC1 A498 cells. *p < 0.05, **p < 0.01, ***p < 0.001.

To explore the effect of DEPDC1 on the malignant biological behavior of RCC, we used HK-2 as a control group, and found that DEPDC1 expression was generally higher in RCC cells, particularly in OS-RC-2 and 786-O cells, while A498 and ACHN cells exhibited relatively low DEPDC1 expression levels as determined by qRT-PCR and western blot (Fig. 3a, b). DEPDC1 knockdown cells (OS-RC-2 and 786-O) and DEPDC1 overexpression cells (A498 and ACHN) were constructed and detected for efficiency using qRT-PCR and western blot (Figs. 3c, d and S2d, e). The effect of DEPDC1 expression on RCC cell proliferation and invasion was tested through CCK-8 cell proliferation assay, transwell migration and invasion assays. The results showed that the proliferation, migration and invasion abilities of RCC cells in the DEPDC1 knockdown group were significantly reduced (Figs. 3e, f and S2f, g), while those in the DEPDC1 overexpression group were significantly increased (Fig. 3g–l). We then constructed a nude mouse subcutaneous tumor model using A498 cells (Vector and OE-DEPDC1) to explore the effect of overexpressing DEPDC1 on RCC cell growth in vivo. The results demonstrated that the subcutaneous tumors in OE-DEPDC1 group were larger with a faster growth rate as compared with those in the Vector group. There was no significant change in body weight of the nude mice between the two groups. IHC staining revealed higher expression of DEPDC1 in the tumor cell nucleus of the OE-DEPDC1 group than that in the Vector group (Fig. 3m–q). In summary, these results show that DEPDC1 promoted the proliferation, migration, invasion and subcutaneous tumor formation ability of RCC cells.

### DEPDC1 is correlated with drug resistance in RCC and enhances glycolysis

**Fig. 4:**
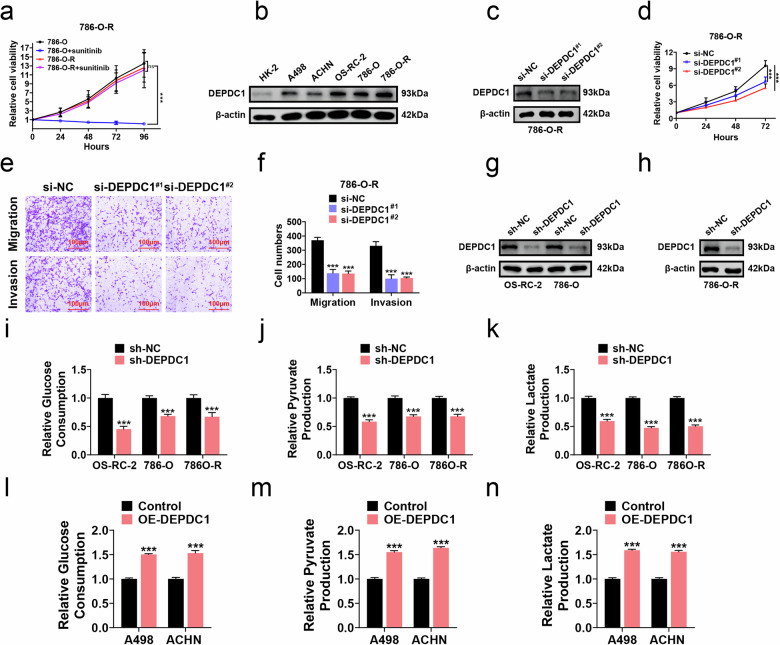
DEPDC1 is correlated with drug resistance and enhances glycolysis in RCC. **a** Cell proliferation assay detects the cell proliferation activity of 786-O cells and 786-O-R cells as well as 786-O cells and 786-O-R cells treated with sunitinib (10 μM). **b** Western blot detecting the protein expression levels of DEPDC1 in HK-2 and five types of RCC cells. **c** Western blot verifying the knockdown efficiency of DEPDC1 in 786-O-R cell. **d** Cell proliferation assay showing changes in the proliferation activity of 786-O-R cells in si-DEPDC1^#1^ and si-DEPDC1^#2^ groups vs si-NC group. **e**, **f** Transwell assay showing the number of migrating and invasive cells of 786-O-R cells in si-DEPDC1^#1^ group and si-DEPDC1^#2^ groups compared to si-NC group, scale bar = 100 μm. **g**, **h** Western blot verifying the knockdown efficiency of DEPDC1 in OS-RC-2 cells, 786-O cells and 786-O-R cells. **i**–**k** Glycolysis function assay showing changes in glucose consumption, pyruvate production, and lactate production of OS-RC-2, 786-O and 786-O-R cells between sh-DEPDC1 and sh-NC groups. **l**–**n** Glycolysis function assay showing changes in glucose consumption, pyruvate production, and lactate production of A498 and ACHN cells between OE-DEPDC1 and Vector groups. *p < 0.05, **p < 0.01, ***p < 0.001.

To investigate the potential reversal of RCC resistance to sunitinib by knocking down DEPDC1, sunitinib-resistant 786-O cell (786-O-R) was established by using CCK8 assays (Fig. 4a). Western blot analysis revealed elevated DEPDC1 protein levels in 786-O-R cells compared to other RCC cells and HK-2 cell (Fig. 4b). DEPDC1 knockdown in 786-O-R cells was confirmed by qRT-PCR and western blot (Figs. 4c and S2h). Subsequent CCK8 assays demonstrated that DEPDC1 knockdown inhibited the proliferation of 786-O-R cells compared with si-NC group (Fig. 4d). Transwell assays further showed reduced migration and invasion in 786-O-R cells with DEPDC1 knockdown (si-DEPDC1^#1^ and si-DEPDC1^#2^) compared to si-NC group (Fig. 4e, f). Stable DEPDC1 knockdown in OS-RC-2, 786-O, and 786-O-R cells was validated using qRT-PCR and western blot (Figs. 4g, h and S2i, j). Additionally, we examined the metabolite content in the cell culture supernatant. Glucose consumption, pyruvate production, and lactate production were measured in DEPDC1 knockdown or overexpression RCC cells, revealing that the glycolytic activity was reduced after DEPDC1 knockdown, and increased after DEPDC1 overexpression (Fig. 4i–n). These findings collectively suggest that DEPDC1 played a role in RCC TKI resistance and glycolysis promotion.

### DEPDC1 regulates RCC glycolysis through AKT/mTOR/HIF1α pathway

**Fig. 5:**
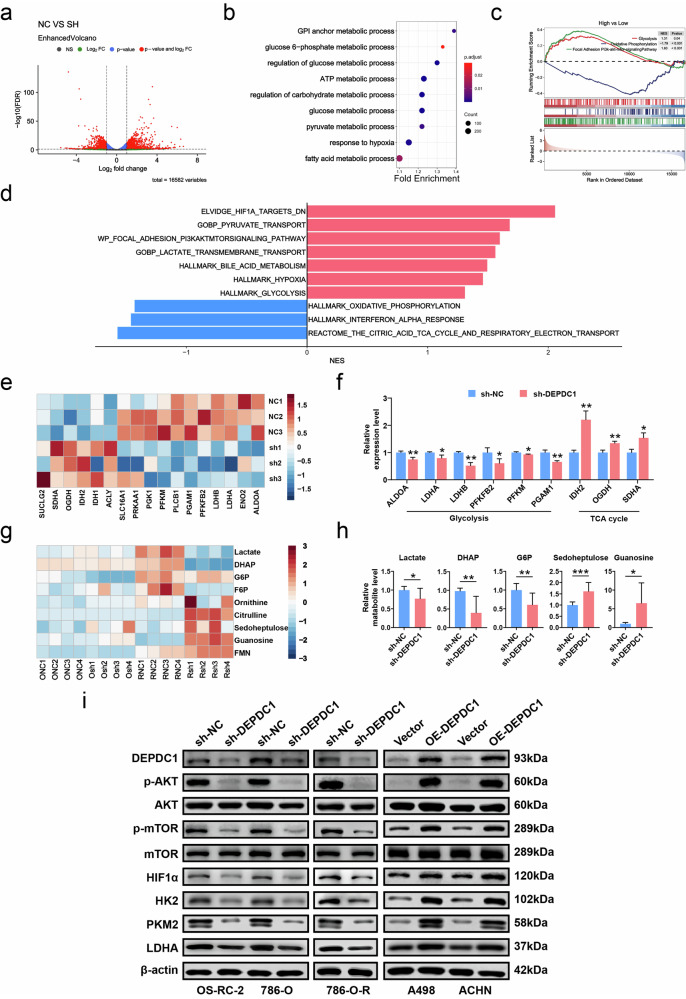
RNA-seq and non-targeted metabolomics reveal that DEPDC1 positively regulates glycolysis in RCC. **a** Volcano plot showing all DEGs sequenced in the RNA-seq of 786-O cells (sh-NC and sh-DEPDC1 groups, *n* = 3). **b** GSEA bubble chart shows the biological processes involved in the DEGs of 786-O cells. **c**, **d** GSEA analysis showing that high expression of DEPDC1 was positively correlated with glycolysis and PI3K/AKT/mTOR and other pathways, and negatively correlated with TCA cycle and oxidative phosphorylation. **e** Heat map showing the expression changes of genes related to the regulation of glycolysis and TCA cycle after knockdown of DEPDC1 in 786-O cells. **f** Histogram showing significantly changed genes in 786-O cells after knocking down DEPDC1. **g** Heat map showing changes in glycolytic and TCA cycle metabolites in 786-O and 786-O-R cells after knocking down DEPDC1. **h** Histogram showing significantly changed glycolytic and TCA cycle metabolites in 786-O and 786-O-R cells after knocking down DEPDC1. **i** Western blot reveals changes in protein expression of AKT/mTOR/HIF1α pathway and key glycolysis enzymes in RCC cells after knocking down or overexpressing DEPDC1. *p < 0.05, **p < 0.01, ***p < 0.001.

DEPDC1, a glycolysis-related gene, has been shown to promote the progression of oral squamous cell carcinoma (OSCC), predict the progression and prognosis of RCC, and serve as a predictor of prognostic risk in hepatocellular carcinoma (HCC) [10, 11, 27]. However, the specific molecular mechanism through which DEPDC1 enhances tumor glycolysis had not yet been fully elucidated. To investigate the molecular mechanism of DEPDC1 in regulating glycolysis in RCC, we conducted transcriptional analysis in 786-O cells (sh-NC and sh-DEPDC1 groups). DEGs revealed by volcano plot are shown in Fig. 5a. Subsequent GSEA results indicated that these DEGs were primarily involved in regulating the glucose metabolic process, pyruvate metabolic process, and hypoxia response, among other processes (Fig. 5b). It was found that the glycolysis, hypoxia and phosphatidylinositol 3-kinase (PI3K)/AKT/mTOR pathways, as well as pyruvate transport was positively regulated in high-expression DEPDC1 group, while the oxidative phosphorylation and TCA cycle pathways were negatively regulated (Fig. 5c, d). Our further analysis focused on the impact of DEPDC1 knockdown on genes involved in glycolysis regulation. The heat map in Fig. 5e shows the relative expression of genes related to glycolysis and TCA cycle. Knocking down DEPDC1 down-regulated ALDOA, LDHA, LDHB, PFKFB2, PFKM and PGAM1 genes, which was associated with glycolysis, while up-regulation of IDH2, OGDH and SDHA genes was associated with TCA cycle (Fig. 5f). The result of GEPIA2 database analysis showed that the expressions of key enzymes (ALDOA, ENO1, GLUT1, GLUT3, HK-2, LDHA, PDK1 and PKM) in glycolysis were significantly increased in KIRC compared with those in normal tissues. Additionally, these enzymes exhibited a strong correlation with DEPDC1 (Fig. S3). To investigate the effect of knocking down DEPDC1 on RCC glycolytic metabolites, LC/MS non-targeted metabolism analysis was conducted on 786-O cells and 786-O-R cells (sh-NC group and sh-DEPDC1 group). The heat map indicated a decrease in the production of glycolytic metabolites, such as lactate, dihydroxyacetone phosphate (DHAP), glucose hexaphosphate (G6P) in RCC cell lines, while there was an increase in TCA cycle metabolites such as sedoheptulose, citrulline, particularly in 786-O-R cells (Fig. 5g). As previous study has shown that Warburg effect caused by VHL mutations also increases TKI resistance of ccRCC, and the difference in glycolytic metabolites is more significant in 786-O-R cells [9]. Specifically, the alterations in glycolytic metabolites and TCA cycle metabolites in Fig. 5h were statistically significant. According to the findings of transcriptomic sequencing, we investigated how DEPDC1 regulated RCC glycolysis by western blot analysis. The results revealed that knocking down DEPDC1 led to a significant reduction in the protein expression of p-AKT, p-mTOR, HIF1α, HK2, PKM2, and LDHA, while overexpression of DEPDC1 resulted in a notable increase in these proteins (Fig. 5i). In conclusion, DEPDC1 acted as a positive regulator of RCC glycolysis via the AKT/mTOR/HIF1α pathway.

### DEPDC1 is associated with poor prognosis and predicts metastasis in RCC

**Fig. 6:**
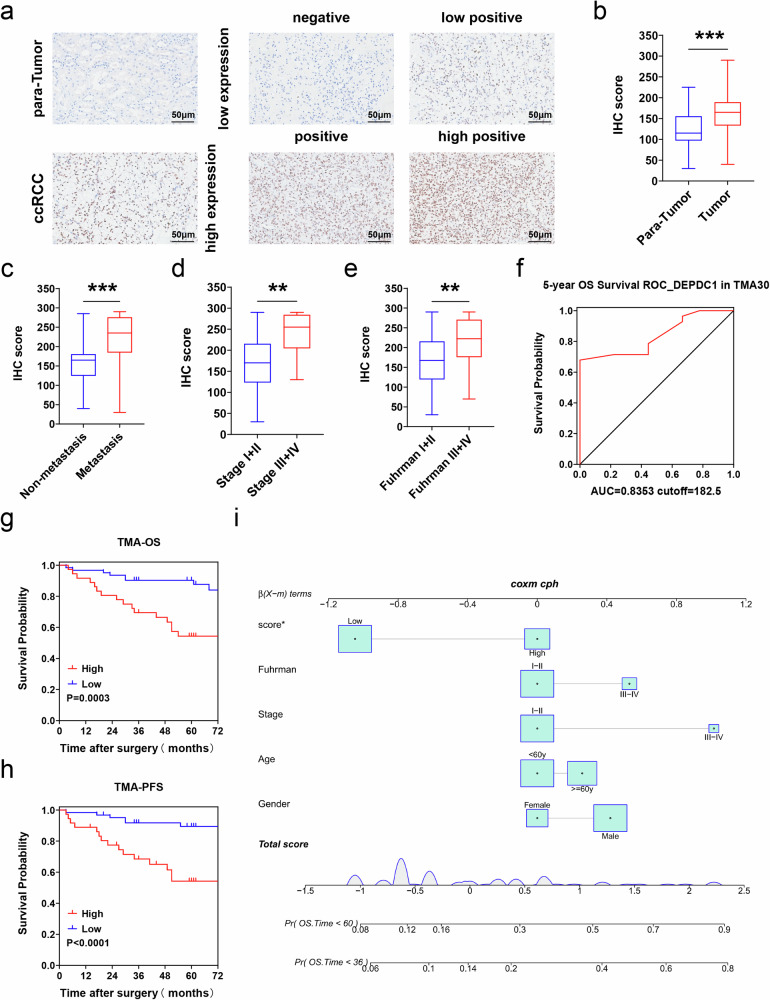
DEPDC1 predicts poor prognosis and metastasis in RCC within our clinical cohort. **a** Representative IHC staining and H-score of DEPDC1 in RCC tissue and paired para-cancerous tissue (TMA2021, *n* = 70), scale bar = 50 μm. **b** IHC scores of RCC tissue and paired para-cancerous tissue (TMA2021, *n* = 70). **c** IHC scores of tumor tissues from patients with and without metastasis. **d** IHC scores of patients with stage I, II and stage III, IV. **e** IHC scores for patients with Fuhrman grades I, II and III, IV. **f** ROC curve of DEPDC1 expression in TMA30 cohort with 5-year OS. **g** K–M survival curve shows OS of DEPDC1 high-expression group and low-expression group. **h** K-M survival curve shows PFS of the DEPDC1 high-expression and low-expression groups. **i** Multivariate nomogram analysis of 3 and 5-year OS. *p < 0.05, **p < 0.01, ***p < 0.001. TMA tissue microarray.

To further explore the significance of DEPDC1 in RCC progression, we conducted a study involving two cohorts (TMA30, *n* = 28; TMA2021, *n* = 70). Quantitative analysis of the TMA2021 cohort using H-score revealed higher DEPDC1 expression in tumor tissues compared to para-cancerous tissues (Fig. 6a, b). Subsequently, we stratified the combined cohorts (TMA30 + TMA2021) based on clinical data and observed higher H-scores in patients with metastasis (Fig. 6c), advanced tumor stage (Fig. 6d), and high Fuhrman grade (Fig. 6e). ROC analysis on the TMA30 cohort for 5-year OS determined an optimal cutoff value for DEPDC1 H-score at 182.5 with an AUC of 0.8353 (Fig. 6f). Further analysis using K-M survival plots demonstrated that both OS and PFS were shorter in patients of high DEPDC1 expression group (Fig. 6g, h). Multivariate Cox nomogram analysis confirmed DEPDC1 expression as an independent risk factor for OS and PFS in RCC patients (Figs. 6i and S4a–c). In summary, our findings suggest that DEPDC1 was closely linked to poor prognosis and could serves as a valuable predictor of RCC metastasis.

## Discussion

As RCC advances, its glycolytic metabolic activity increases, potentially contributing to resistance against TKIs due to VHL gene mutation [7, 9]. Thus, targeting glycolysis could offer a dual benefit of inhibiting RCC progression and reversing TKI resistance. A recent study has highlighted the role of phosphoglycerate kinase 1 (PGK1) in promoting tumorigenesis and sorafenib resistance in ccRCC by activating the CXCR4/ERK pathway and enhancing glycolysis [28]. Additionally, CircME1 has been implicated in promoting aerobic glycolysis and sunitinib resistance in ccRCC through cis-regulating ME1 [29]. However, existing research on the impact of glycolysis on RCC progression and drug resistance is limited, our study is the first to identify glycolysis as a crucial factor associated with poor prognosis and drug resistance in RCC patients by utilizing scRNA-seq.

After clarifying the research focus on glycolysis, we identified DEPDC1 as the glycolysis-related gene with the highest hazard ratio (HR) in TCGA-KIRC and the glycolysis gene set (HR = 3.023, *p* < 0.001) (Fig. S2a). DEPDC1, initially identified in bladder cancer, was found to be overexpressed in various cancers including bladder, liver, and lung cancer, and is widely recognized as an oncogene [30–32]. Although some previous studies on RCC have primarily incorporated DEPDC1 in glycolysis risk prognostic models, they lacked in-depth in vitro and in vivo experimental investigations [27]. In OSCC, DEPDC1 as a glycolysis-related gene has been shown to enhance aerobic glycolysis, migration, and invasion via the WNT/β-Catenin pathway [11]. Furthermore, DEPDC1 has been implicated in promoting aggressive features in breast cancer by activating the PI3K/AKT/mTOR pathway [33].

Consequently, the association between DEPDC1 and RCC progression as well as drug resistance warrants further exploration. In this study, we observed that the sensitivity to sunitinib and pazopanib was reduced in high DEPDC1 group vs low DEPDC1 group using the GDSC database (Fig. 2f, g). GSEA revealed a positive correlation between high DEPDC1 expression and glycolysis (Fig. 2l, m). Additionally, we established sunitinib-resistant RCC cells (786-O-R) and performed in vitro experiment, finding that elevation of the DEPDC1 expression promoted glycolysis and drug resistance in RCC (Fig. 4).

A recent proteomic study on ccRCC by Ye’s team at Fudan University Shanghai Cancer Center revealed that the PI3K/AKT/mTOR pathway was the mechanism contributing to sunitinib resistance in ccRCC patients, which is consistent with our RNA-seq findings [9]. Our study indicated a positive correlation of high DEPDC1 expression with glycolysis and the PI3K/AKT/mTOR pathway, and a negative correlation with oxidative phosphorylation and TCA cycle (Fig. 5b–d). Previous studies have demonstrated that the PI3K/AKT pathway activated mTOR, leading to the upregulation of glycolytic enzymes through the transcription factor HIF1α [34, 35]. Our RNA-seq results showed that knocking down DEPDC1 could downregulate key glycolytic enzymes like ALDOA [36], LDHA [37], LDHB [38], and PFKFB2 [39], while upregulating enzymes from the TCA cycle such as OGDH [40] and SDHA [41] (Fig. 5f). Western blot analysis demonstrated that DEPDC1 activated mTOR signaling, upregulated HIF1α, and increased the expression of glycolytic enzymes HK2, PKM2, and LDHA (Fig. 5i). Previous metabolomic studies also support our findings, showing increased glycolytic metabolites in sunitinib-treated RCC-resistant cells vs untreated RCC cells [42]. Our non-targeted metabolomics further revealed more pronounced differences in glycolytic and TCA cycle metabolites upon DEPDC1 knockdown in 786-O-R cells vs 786-O cells (Fig. 5g, h). Therefore, our study strongly suggests that the glycolysis-related gene DEPDC1 promotes glycolysis and TKI resistance in RCC by activating the AKT/mTOR/HIFα pathway.

## Conclusion

**Fig. 7:**
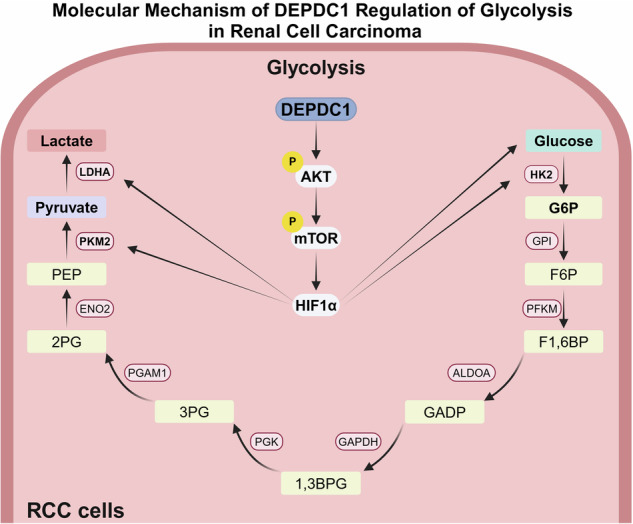
Molecular mechanism of DEPDC1 in RCC.

In summary, our study is the first to utilize scRNA-seq to reveal that glycolysis plays a significant role in the poor prognosis and TKI resistance in RCC. Additionally, multi-omics analysis identified that DEPDC1 could promote RCC glycolysis and TKI resistance via the AKT/mTOR/HIFα pathway (Fig. 7). Clinical findings from our cohort indicate that high DEPDC1 expression is correlated with poor prognosis and metastasis prediction in RCC patients. Overall, our research presents a novel therapeutic target in glucose metabolism for treating advanced TKI-resistant RCC patients. However, our study still has certain limitations. The next step is to explore how DEPDC1 regulates the AKT/mTOR/HIF1α pathway and key glycolytic enzymes, and to verify the effects of DEPDC1 in primary drug-resistant tumor animal models of renal cell carcinoma.

### Supplementary information

supplementary figure and table legends

Fig S1

Fig S2

Fig S3

Fig S4

Table S1

Table S2

Table S3

Table S4

Table S5

Table S6

Table S7

Table S8

Table S9

Uncropped Western Blots

## Data Availability

The data and material during the current study were available from the corresponding author on reasonable request.
