# Supplementary material for: DEPDC1 as a metabolic target regulates glycolysis in renal cell carcinoma through AKT/mTOR/HIF1α pathway
Source: Cell Death Dis. 2024 Jul 27;15(7):533. doi: 10.1038/s41419-024-06913-1 (PMC11283501; doi:10.1038/s41419-024-06913-1)
Supplement: Supplementary file 1 — supplementary figure and table legends [file 41419_2024_6913_MOESM1_ESM.docx]

**Supplementary Materials**

**Fig. S1. a,** Optimal soft threshold**. b,** hME showing the module characteristic values ​​of gene modules in different clinical stages**. c,** kME showing the correlation between core genes and gene modules.

**Fig. S2. a,** Univariate Cox regression analysis of top 19 genes and clinical information. **b,** OS of DEPDC1 high and low groups in GEPIA2. **c,** DFS of DEPDC1 high and low groups in GEPIA2**. d,** qRT-PCR showing the knockdown efficiency of DEPDC1 in OS-RC-2 and 786-O cells. **e,** qRT-PCR showing the overexpression efficiency of DEPDC1 in A498 cells and ACHN cells. **f, g,** Transwell assay showing changes in the number of migration and invasion cells in OS-RC-2 cells and 786-O cells in DEPDC1 knockdown groups (si-DEPDC1^#1^ and si-DEPDC1^#2^) compared with si-NC group, scale bar = 100 μm. **h,** qRT-PCR showing the knockdown efficiency of DEPDC1 in 786-O-R cell. **i, j,** qRT-PCR verifying the stablely knockdown efficiency of DEPDC1 in OS-RC-2 cells, 786-O cells and 786-O-R cells.

**Fig. S3.** Histogram showing the expression levels of key glycolytic enzymes in KIRC, and scatter plot showing the spearman correlation analysis of key glycolytic enzymes and DEPDC1.

**Fig. S4.** **a，**The nomogram for 3-year and 5-year PFS in our cohort. **b，**The calibration plot of nomogram for 3-year and 5-year OS. **c，**The calibration plot of nomogram for 3-year and 5-year PFS.

**Table S1.** Clinical characteristics of patients according to DEPDC1 expression in TMA30 (n=28).

**Table S2.** Clinical characteristics of patients according to DEPDC1 expression in TMA2021 (n=70).

**Table S3**. Clinical characteristics of patients according to DEPDC1 expression in RCC (n=531).

**Table S4.** 200 glycolysis genes from MSigDB.

**Table S5.** sh sequences, OE sequences, si sequences.

**Table S6.** qRT-PCR primer.

**Table S7.** Maker annotations.

**Table S8.** hME Malignant.

**Table S9.** kME Malignant.
