# Supplementary figures and images for: DEPDC1 as a metabolic target regulates glycolysis in renal cell carcinoma through AKT/mTOR/HIF1α pathway
Source: Cell Death Dis. 2024 Jul 27;15(7):533. doi: 10.1038/s41419-024-06913-1 (PMC11283501; doi:10.1038/s41419-024-06913-1)

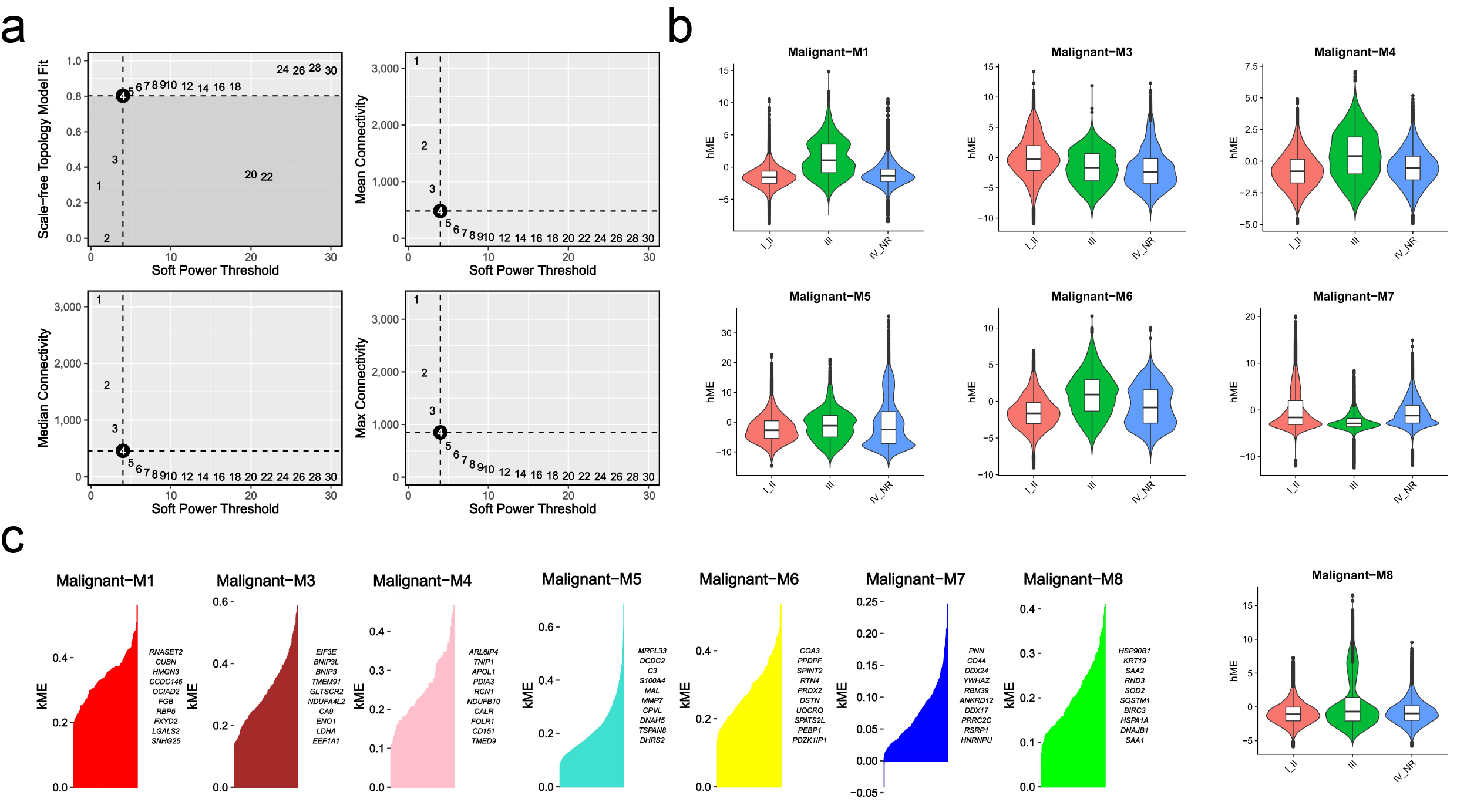

Supplement: Supplementary file 2 — Fig S1 [file 41419_2024_6913_MOESM2_ESM.png]

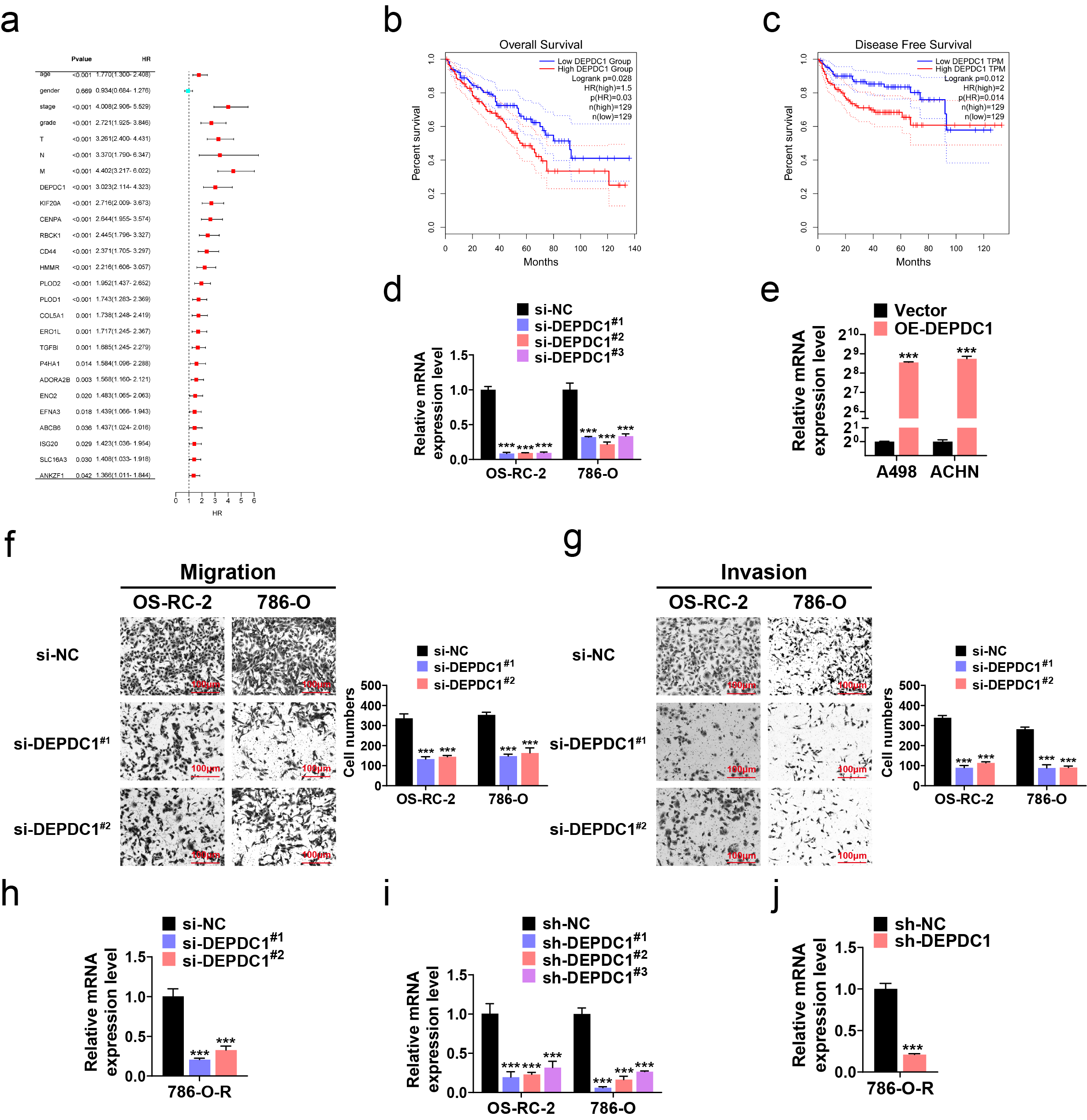

Supplement: Supplementary file 3 — Fig S2 [file 41419_2024_6913_MOESM3_ESM.png]

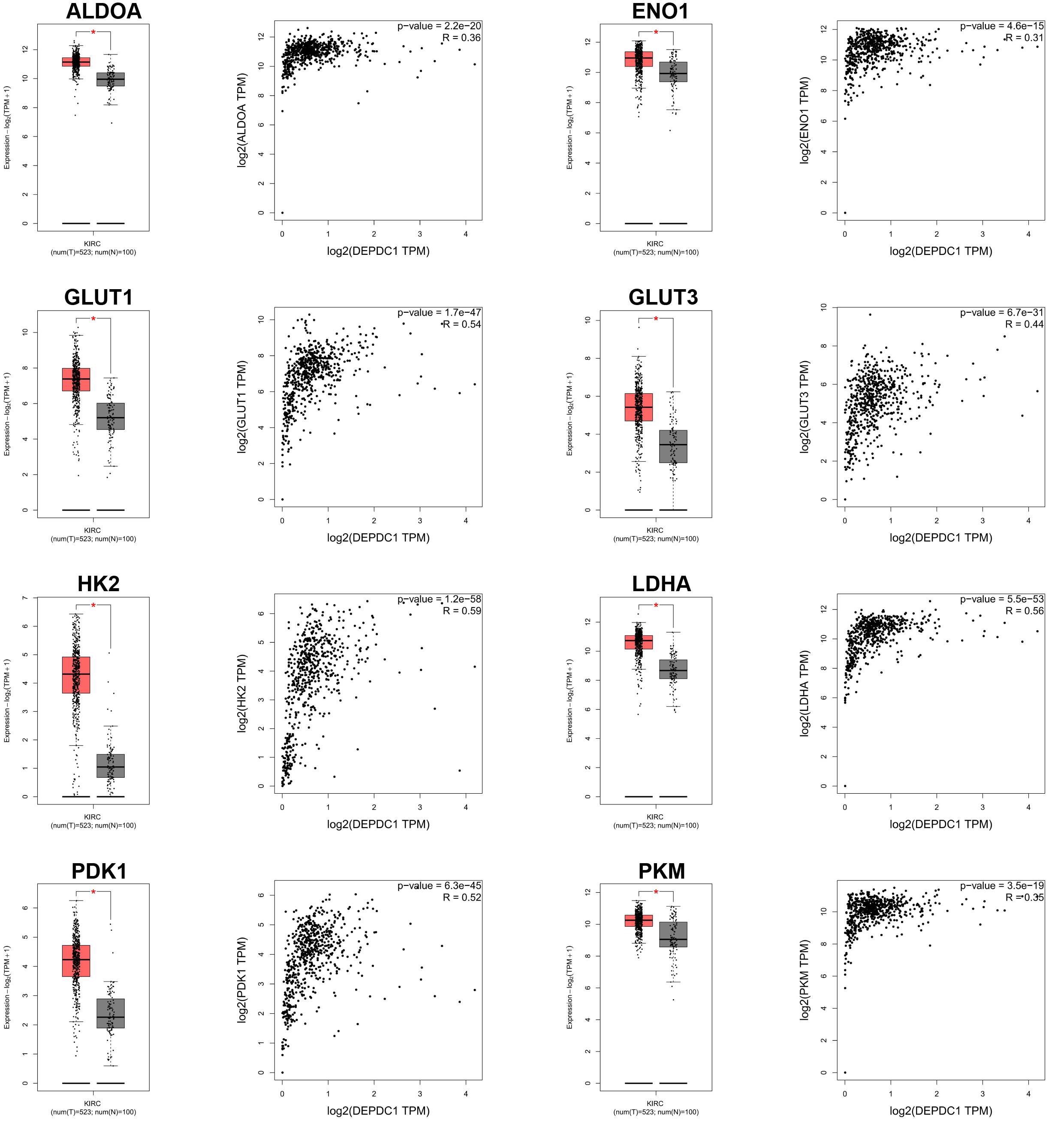

Supplement: Supplementary file 4 — Fig S3 [file 41419_2024_6913_MOESM4_ESM.png]

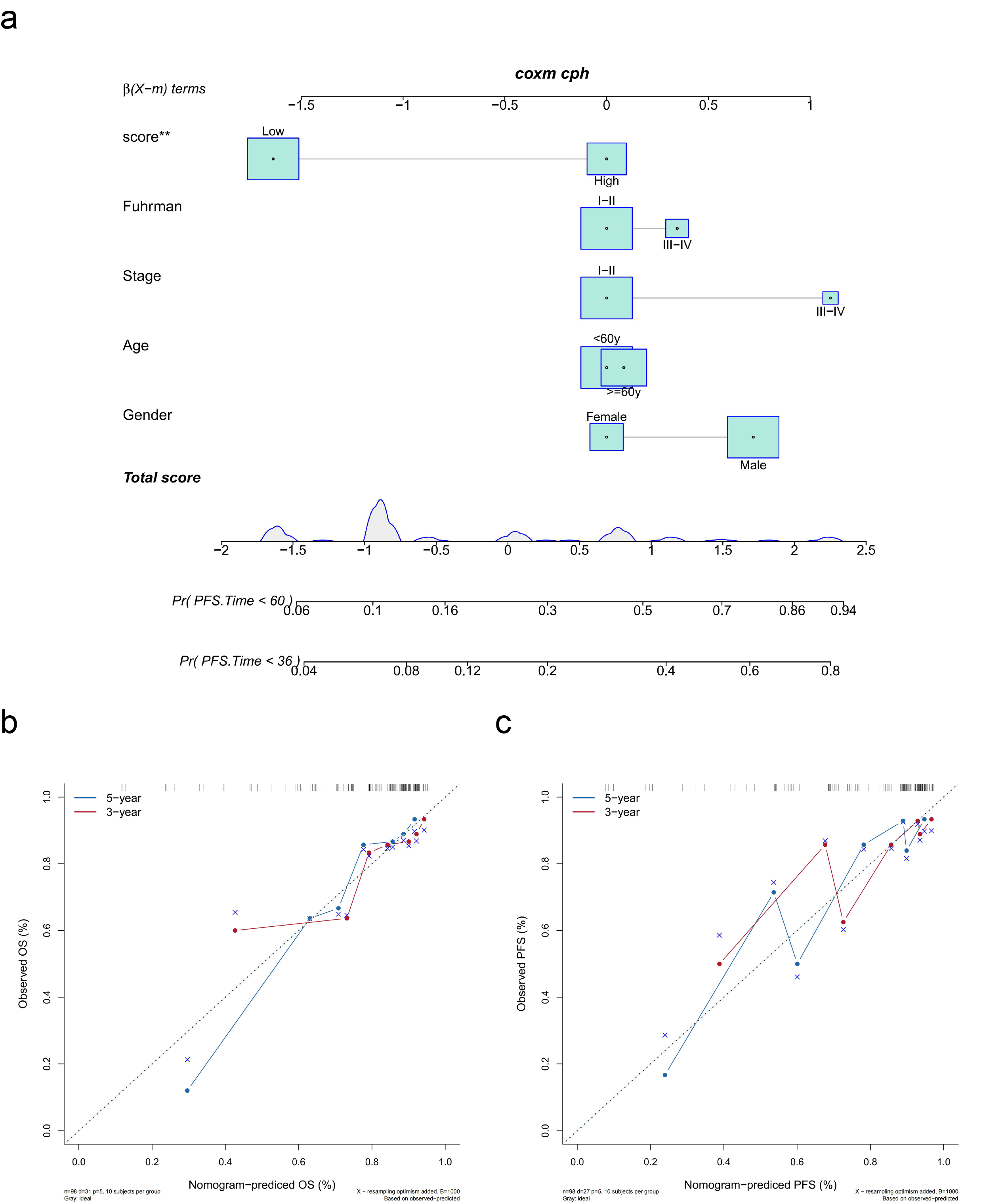

Supplement: Supplementary file 5 — Fig S4 [file 41419_2024_6913_MOESM5_ESM.png]
