# Supplementary material for: DEPDC1 as a metabolic target regulates glycolysis in renal cell carcinoma through AKT/mTOR/HIF1α pathway
Source: Cell Death Dis. 2024 Jul 27;15(7):533. doi: 10.1038/s41419-024-06913-1 (PMC11283501; doi:10.1038/s41419-024-06913-1)
Supplement: Supplementary file 6 — Table S1 [file 41419_2024_6913_MOESM6_ESM.docx]

**Table S1**. Clinical characteristics of patients according to DEPDC1 expression in TMA30 (n=28).

| **Characteristics** | **DEPDC1 in TMA30** | | **Sum(n=28)** | ***P value*** |
| --- | --- | --- | --- | --- |
|  | **High expression(n=19)** | **Low expression(n=9)** |  |  |
| Diagnosis age |  |  |  | 0.2248 |
| <60 | 8 | 6 | 14 |  |
| ≥60 | 11 | 3 | 14 |  |
| Gender |  |  |  | 0.5212 |
| Male | 15 | 8 | 23 |  |
| Female | 4 | 1 | 5 |  |
| Fuhrman grade |  |  |  | 0.0615 |
| G1-2 | 10 | 8 | 18 |  |
| G3-4 | 9 | 1 | 10 |  |
| TNM stage |  |  |  | 0.0572 |
| I-II | 13 | 9 | 22 |  |
| III-IV | 6 | 0 | 6 |  |
| 5-year any metastases or recurrence |  |  |  | 0.0115 |
| Yes | 18 | 5 | 23 |  |
| No | 1 | 4 | 5 |  |
| 5-year overall survival |  |  |  | 0.5747 |
| Dead | 18 | 8 | 26 |  |
| Alive | 1 | 1 | 2 |  |
