# Supplementary material for: DEPDC1 as a metabolic target regulates glycolysis in renal cell carcinoma through AKT/mTOR/HIF1α pathway
Source: Cell Death Dis. 2024 Jul 27;15(7):533. doi: 10.1038/s41419-024-06913-1 (PMC11283501; doi:10.1038/s41419-024-06913-1)
Supplement: Supplementary file 7 — Table S2 [file 41419_2024_6913_MOESM7_ESM.docx]

**Table S2.** Clinical characteristics of patients according to DEPDC1 expression in TMA2021 (n=70)

| **Characteristics** | **DEPDC1 in TMA2021** | | **Sum(n=70)** | ***P value*** |
| --- | --- | --- | --- | --- |
|  | **High expression(n=17)** | **Low expression(n=53)** |  |  |
| Diagnosis age |  |  |  | 0.0251 |
| <60 | 6 | 35 | 41 |  |
| ≥60 | 11 | 18 | 29 |  |
| Gender |  |  |  | 0.0626 |
| Male | 8 | 38 | 46 |  |
| Female | 9 | 15 | 24 |  |
| Fuhrman grade |  |  |  | 0.5888 |
| G1-2 | 15 | 49 | 64 |  |
| G3-4 | 2 | 4 | 6 |  |
| TNM stage |  |  |  | 0.3895 |
| I-II | 16 | 52 | 68 |  |
| III-IV | 1 | 1 | 2 |  |
| Any metastases or recurrence |  |  |  | 0.0149 |
| Yes | 3 | 1 | 4 |  |
| No | 14 | 52 | 66 |  |
| Overall survival |  |  |  | 0.0026 |
| Dead | 4 | 1 | 5 |  |
| Alive | 13 | 52 | 65 |  |
