# Supplementary material for: DEPDC1 as a metabolic target regulates glycolysis in renal cell carcinoma through AKT/mTOR/HIF1α pathway
Source: Cell Death Dis. 2024 Jul 27;15(7):533. doi: 10.1038/s41419-024-06913-1 (PMC11283501; doi:10.1038/s41419-024-06913-1)
Supplement: Supplementary file 8 — Table S3 [file 41419_2024_6913_MOESM8_ESM.docx]

**Table S3**. Clinical characteristics of patients according to DEPDC1 expression in RCC (n=531).

| **Characteristics** | **DEPDC1 in RCC** | | **Sum(n=531)** | ***P value*** |
| --- | --- | --- | --- | --- |
|  | **High expression(n=140)** | **Low expression(n=391)** |  |  |
| Diagnosis age |  |  |  | 0.0639 |
| <60 | 56 | 192 | 248 |  |
| ≥60 | 84 | 199 | 283 |  |
| Gender |  |  |  | 0.1330 |
| Male | 99 | 249 | 348 |  |
| Female | 41 | 142 | 183 |  |
| Pathological grade |  |  |  | <0.0001 |
| G1-2 | 42 | 201 | 243 |  |
| G3-4 | 96 | 184 | 280 |  |
| NA | 2 | 6 | 8 |  |
| T stage |  |  |  | <0.0001 |
| I-II | 65 | 258 | 323 |  |
| III-IV | 73 | 132 | 205 |  |
| NA | 2 | 1 | 3 |  |
| N stage |  |  |  | <0.0001 |
| ＋ | 12 | 5 | 17 |  |
| － | 62 | 177 | 239 |  |
| NA | 67 | 209 | 276 |  |
| M stage |  |  |  | <0.0001 |
| ＋ | 36 | 42 | 78 |  |
| － | 102 | 320 | 422 |  |
| NA | 2 | 29 | 31 |  |
| Overall survival |  |  |  | <0.0001 |
| Dead | 64 | 107 | 171 |  |
| Alive | 76 | 284 | 360 |  |

* DEPDC1 was divided into high and low expression groups with an optimal cutoff value of 0.60

DEPDC1 low expression: Gene expression level of DEPDC1<0.60;

DEPDC1 high expression: Gene expression level of DEPDC1≥0.60;
