# Supplementary material for: DEPDC1 as a metabolic target regulates glycolysis in renal cell carcinoma through AKT/mTOR/HIF1α pathway
Source: Cell Death Dis. 2024 Jul 27;15(7):533. doi: 10.1038/s41419-024-06913-1 (PMC11283501; doi:10.1038/s41419-024-06913-1)

**Figure 3b**

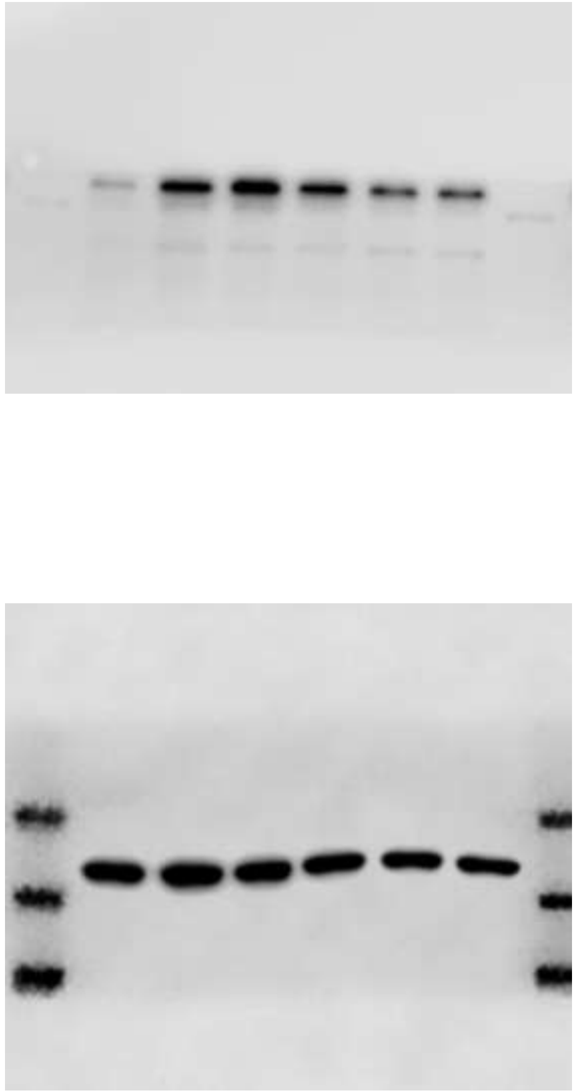

DEPDC1 93kDa

β-actin 42kDa

**Figure 3c**

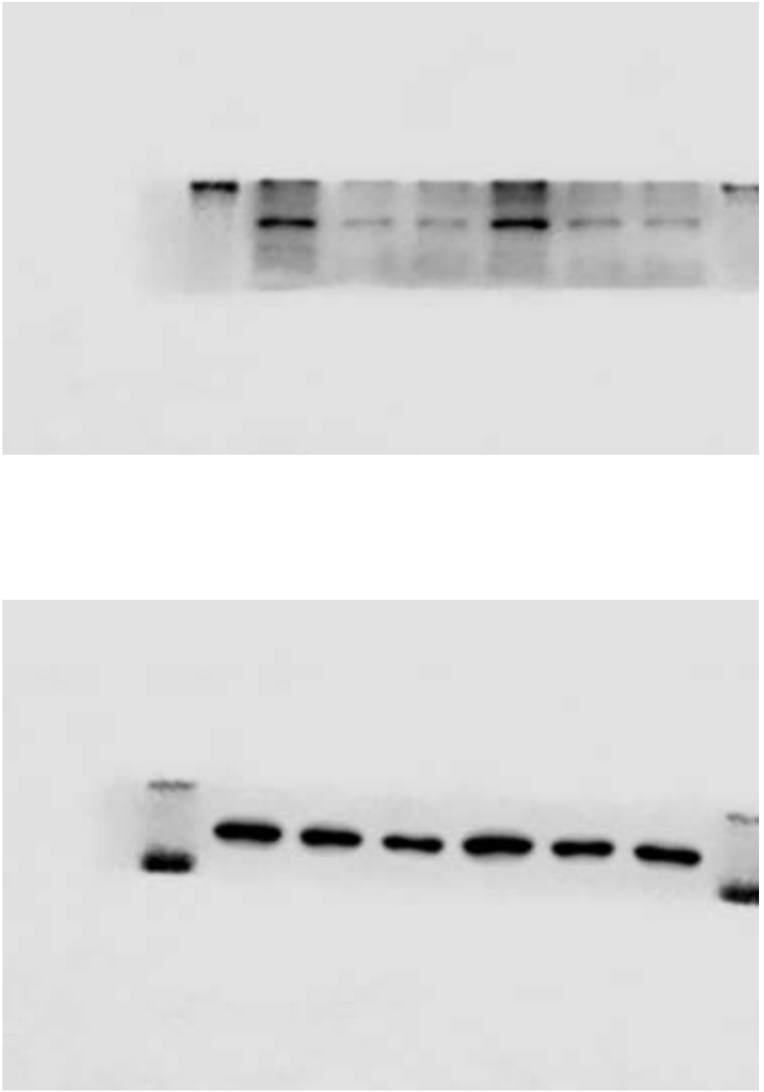

DEPDC1 93kDa

β-actin 42kDa

## Figure 3d

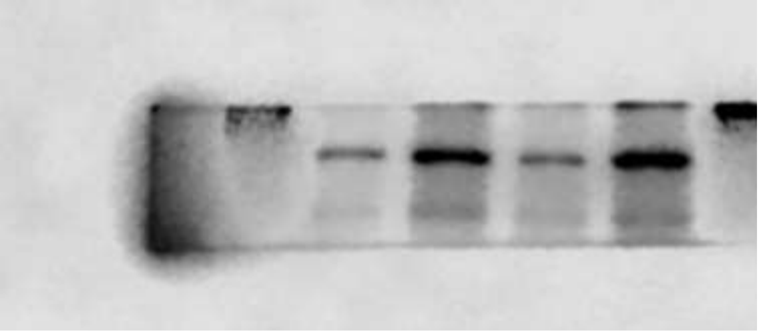

DEPDC1 93kDa

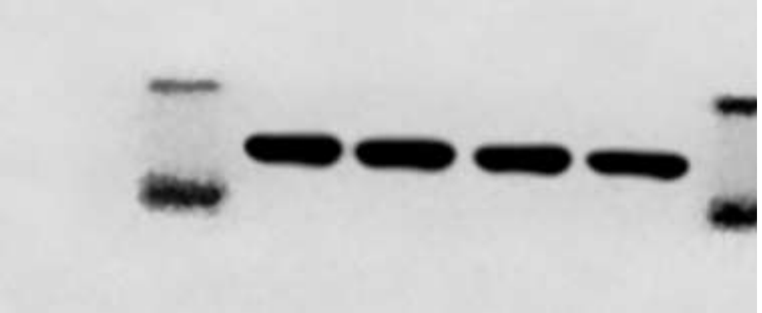

β-actin 42kDa

## Figure 4b

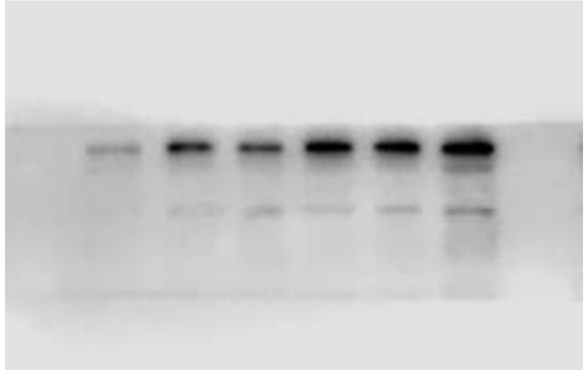

DEPDC1 93kDa

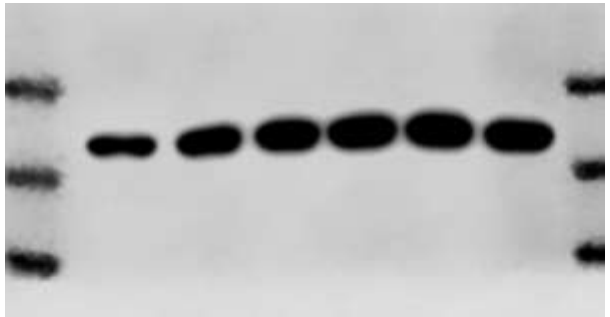

β-actin 42kDa

## Figure 4c

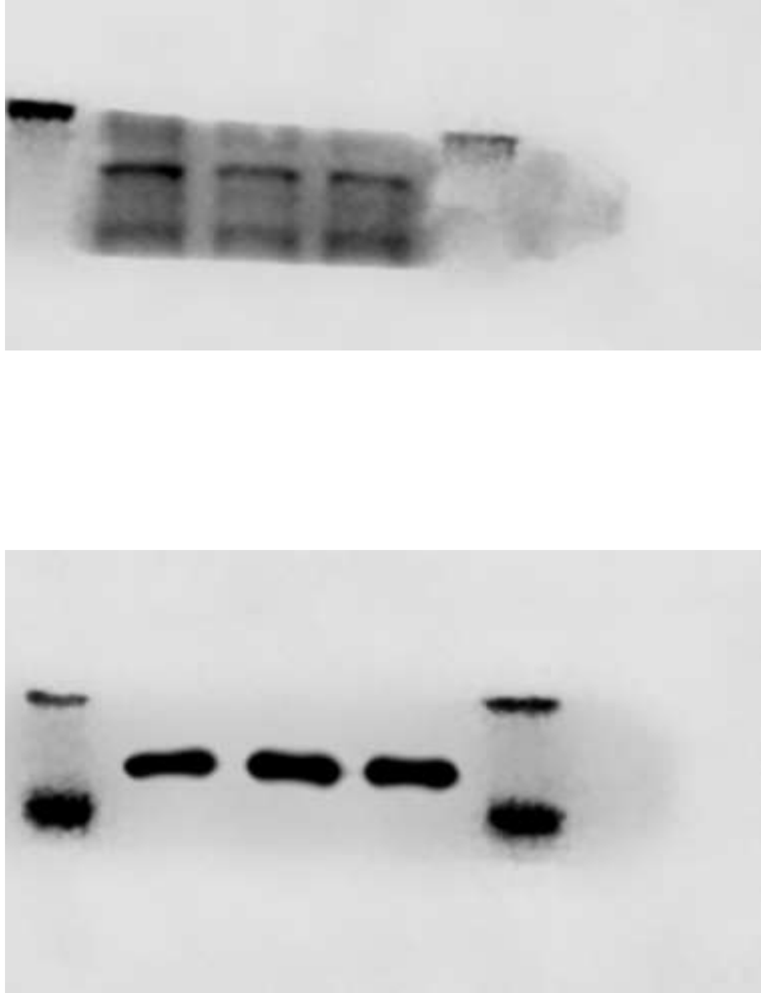

DEPDC1 93kDa

β-actin 42kDa

## Figure 4g

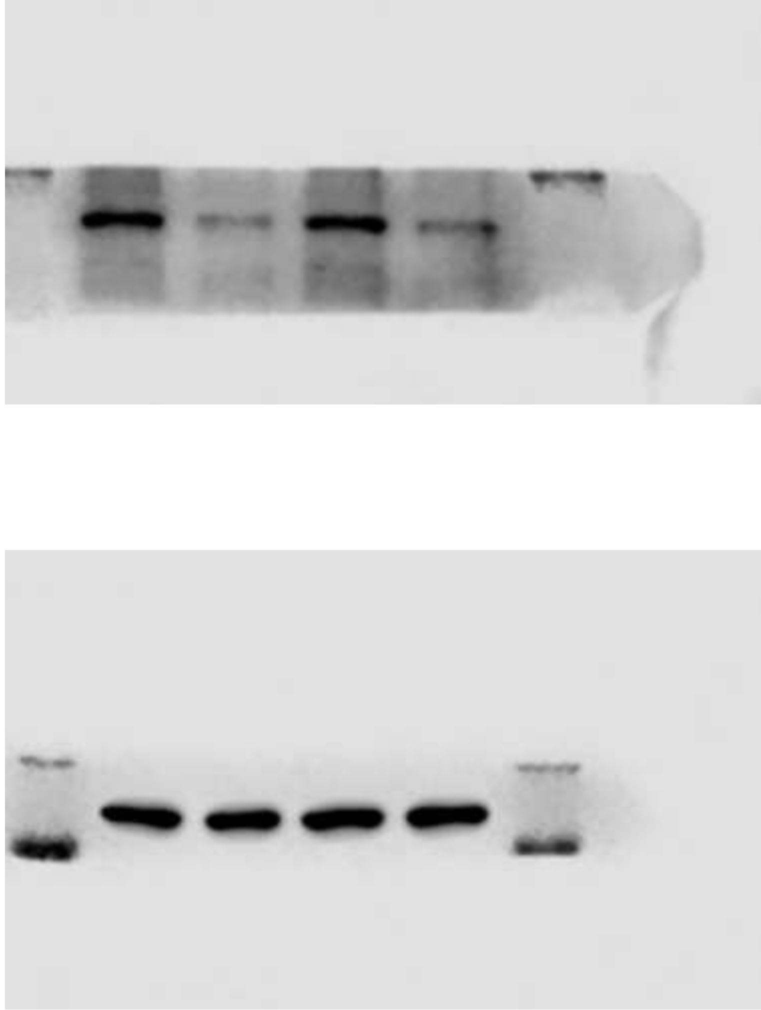

DEPDC1 93kDa

β-actin 42kDa

# Figure 4h

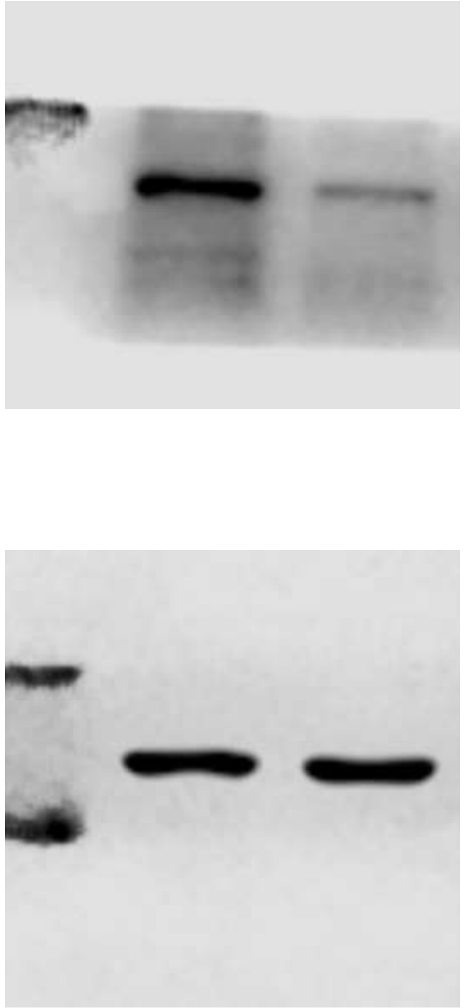

DEPDC1 93kDa

β-actin 42kDa

Figure 5i—OS-RC-2+786-O

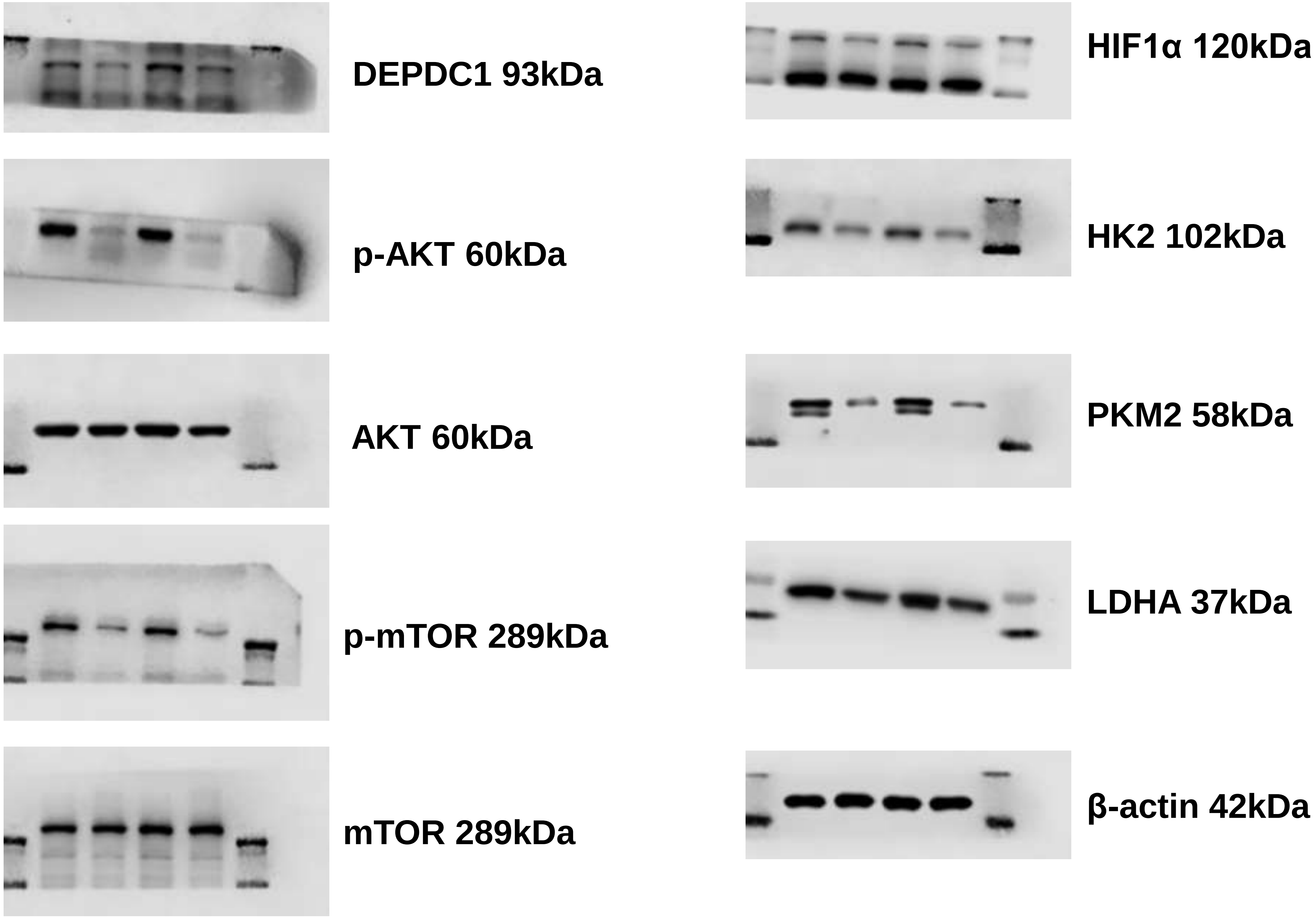

## Figure 5i—786-O-R

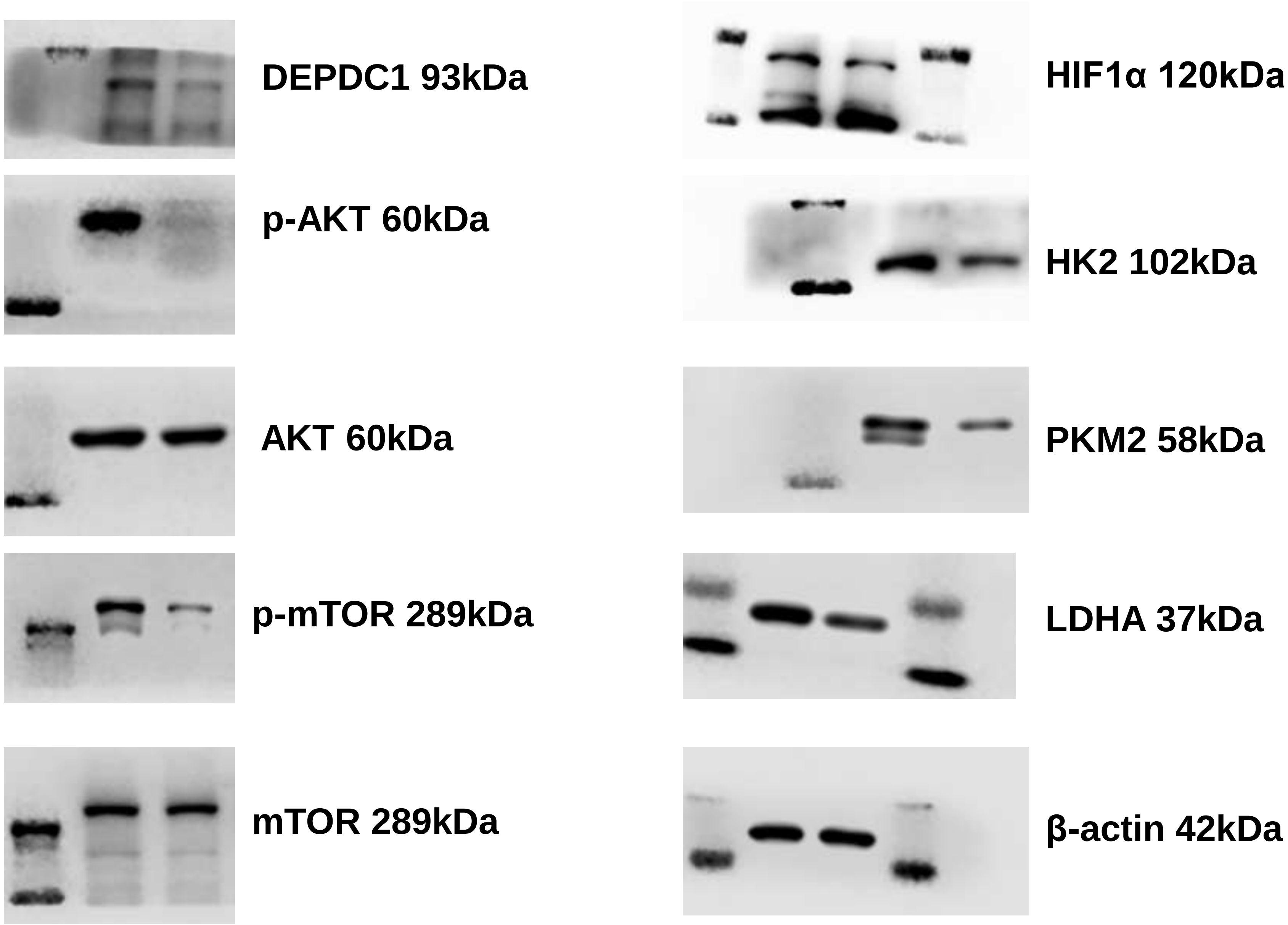

## Figure 5i—A498+ACHN

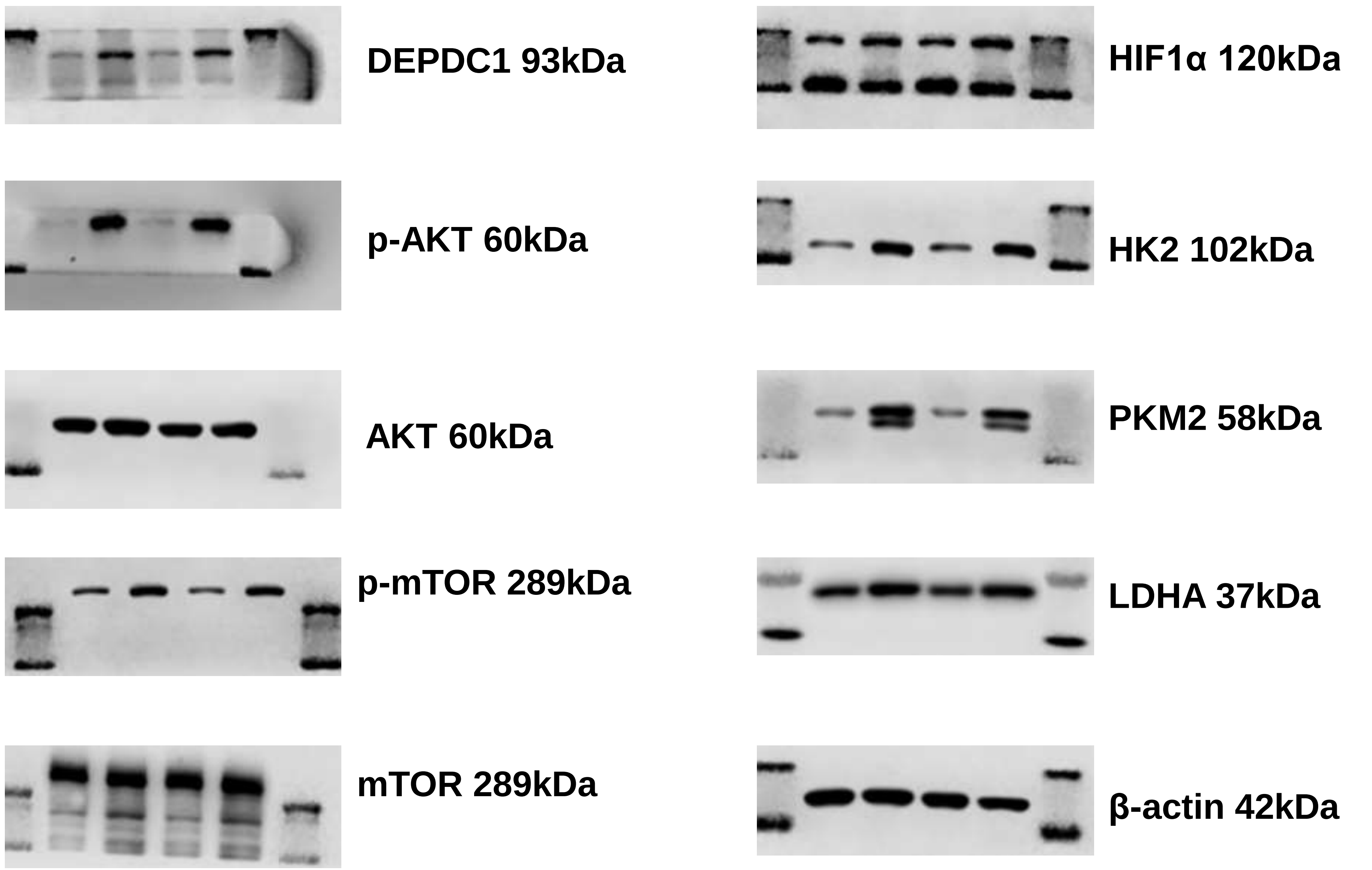

Supplement: Supplementary file 15 — Uncropped Western Blots [file 41419_2024_6913_MOESM15_ESM.pdf]
